# The Specific Alteration of Gut Microbiota in Diabetic Kidney Diseases—A Systematic Review and Meta-Analysis

**DOI:** 10.3389/fimmu.2022.908219

**Published:** 2022-06-17

**Authors:** Yuwei Wang, Jin Zhao, Yunlong Qin, Zixian Yu, Yumeng Zhang, Xiaoxuan Ning, Shiren Sun

**Affiliations:** ^1^ Department of Postgraduate Student, Xi’an Medical University, Xi’an, China; ^2^ Department of Nephrology, Xijing Hospital, Fourth Military Medical University, Xi’an, China; ^3^ Department of Nephrology, Bethune International Peace Hospital, Shijiazhuang, China; ^4^ Department of Geriatric, Xijing Hospital, Fourth Military Medical University, Xi’an, China

**Keywords:** diabetic kidney diseases, gut microbiota, gut dysbiosis, meta-analysis, systematic review

## Abstract

**Background:**

Emerging evidence indicates that gut dysbiosis is involved in the occurrence and development of diabetic kidney diseases (DKD). However, the key microbial taxa closely related to DKD have not been determined.

**Methods:**

PubMed, Web of Science, Cochrane, Chinese Biomedical Databases, China National Knowledge Internet, and Embase were searched for case-control or cross-sectional studies comparing the gut microbiota of patients with DKD and healthy controls (HC) from inception to February 8, 2022, and random/fixed-effects meta-analysis on the standardized mean difference (SMD) were performed for alpha diversity indexes between DKD and HC, and beta diversity indexes and the relative abundance of gut microbiota were extracted and summarized qualitatively.

**Results:**

A total of 16 studies (578 patients with DKD and 444 HC) were included. Compared to HC, the bacterial richness of patients with DKD was significantly decreased, and the diversity indexes were decreased but not statistically, companying with a distinct beta diversity. The relative abundance of phylum *Proteobacteria*, *Actinobacteria*, and *Bacteroidetes*, family *Coriobacteriaceae*, *Enterobacteriaceae*, and *Veillonellaceae*, genus *Enterococcus*, *Citrobacter*, *Escherichia*, *Klebsiella*, *Akkermansia*, *Sutterella*, and *Acinetobacter*, and species *E. coli* were enriched while that of phylum *Firmicutes*, family *Lachnospiraceae*, genus *Roseburia*, *Prevotella*, and *Bifidobacterium* were depleted in patients with DKD.

**Conclusions:**

The gut microbiota of patients with DKD may possess specific features characterized by expansion of genus *Escherichia*, *Citrobacter*, and *Klebsiella*, and depletion of *Roseburia*, which may contribute most to the alterations of their corresponding family and phylum taxa, as well as the bacterial diversity and composition. These microbial taxa may be closely related to DKD and serve as promising targets for the management of DKD.

**Systematic Review Registration:**

https://www.crd.york.ac.uk/prospero/, identifier CRD42021289863.

## Introduction

Data released by the International Diabetes Federation (IDF) in 2021 show that about 537 million adults aged 20-79 are diagnosed with diabetes worldwide, which will reach 783 million by 2045 (1). About 20%-40% of diabetics can develop diabetic kidney disease (DKD) (2, 3). DKD is the most common microvascular complication of diabetes, and also the primary cause of end-stage renal disease (ESRD) (4), and its all-cause mortality is approximately 30 times higher than that in diabetic patients without nephropathy (5), which brings a huge economic burden to patients and the country. The pathogenesis of critical pathological damage, including glomerular hypertrophy, mesangial expansion, tubulointerstitial fibrosis, and inflammation in DKD, has not been fully elucidated (6), and traditional treatments such as blood pressure lowering, glucose-lowering, and lipid regulation have proven incapable of stopping progressive kidney damage (7). Hence, the specific pathogenic mechanisms should be determined for better management of DKD.

The human gut microbiome was described as the “second genome” that controls human health (8), which affects human metabolic and immune functions through genes, intermediates, and metabolic activity. Recent studies suggest that lower alpha-diversity and the characteristic of a pro-inflammatory environment in gut microbiota dysbiosis promote insulitis by activating and proliferating pancreatic‐draining lymph node T cells, particularly diabetogenic CD8+ T cells, which may contribute to the risk of developing type 1 diabetes (T1D) in genetically predisposed individuals (9). In the case of type 2 diabetes (T2D), gut microbial taxa can serve as a biomarker for disease diagnosis (10) and predicting remission (11), which interact with dietary constituents to modulate inflammation, affect gut permeability, glucose and lipid metabolism, insulin sensitivity, and overall energy homeostasis, involving in the pathophysiology of T2D (12). DKD is one of the most common and serious complications of diabetes, and the emerging role of its gut microbiota has attracted wide attention and is considered an important factor affecting the occurrence and development of DKD. Studies have shown that the gut microbiome mediated the synthesis, modification, and uptake of bile acids *via* activation of key nuclear receptors (such as the G protein-coupled receptor TGR5 and the farnesoid X receptor) (13–15), and the production of glucagon-like peptide 1 (GLP-1) and peptide YY (PYY) (16) to maintain energy balance and glucose homeostasis. In the disturbed gut microbiota of patients with DKD, overexpression of Gram-negative bacteria is accompanied by markedly elevated serum levels of the potent immunostimulant lipopolysaccharide (LPS), which correlates with inflammatory markers, such as interleukin 6 (IL-6), C-reactive protein (CRP), and tumor necrosis factor-α (TNFα) (17). Meanwhile, the expansion of bacteria containing urease leads to increased ammonia concentrations and pH in the intestinal lumen, leading to enhanced intestinal permeability, allowing substances such as indole and p-cresol to be transferred to the blood and metabolized into uremic toxins, which induce kidney damage (18). In addition, gut microbiome dysbiosis can lead to the thickened glomerular basement membrane and podocyte foot process effacement by activating the renin-angiotensin system (19), and tubulointerstitial injury *via* the disruption of cholesterol homeostasis (20) to promote the progression of DKD in preclinical models. While gut microbiota interventions such as probiotics supplementation (21, 22), antibiotic administration (23, 24), and fecal microbiota transplantation (24) can partially improve renal function and pathology in DKD. These evidences suggested that intervention targeting specific microbial taxa of DKD may be an effective strategy for the prevention and treatment of DKD.

Although a large number of case-control studies or cross-sectional studies in different populations have characterized the gut microbiota of DKD, inconsistent results describing the microbial differences have been reported between DKD and healthy controls (HC). For example, Xin Xiaohong et al. (25) found a significantly higher alpha diversity in DKD compared with HC, whereas Xuguang Bao et al. (26) revealed the alpha diversity in DKD decreased significantly. On the other hand, changes in the abundance of genus *Lachnospira (* 26, 27), *Rothia (* 25, 27), and *Lachnoclostridium (* 25, 28) were reversed in two studies of DKD compared with HC. In short, the gut microbiota differences between DKD and HC lack obvious reproducibility in different studies when identifying the gut microbiome characteristics in DKD. Therefore, we conducted a systematic review and meta-analysis to dissect the differences of bacterial diversity, the relative abundance of microbial taxa, and bacterial metabolites in the gut microbiota between patients with DKD and HC to identify the crucial taxa that are closely related to DKD and provide potential bacterial targets for the prevention and treatment of DKD.

## Methods

The scheme of the review has been pre-registered in PROSPERO (CRD42021289863). We followed the Preferred Reporting Items for the Systematic Reviews and Meta-analyses (PRISMA) reporting guidelines (29).

### Search Strategy

We conducted extensive literature searches in PubMed, Web of Science, Cochrane, Chinese Biomedical Databases (CBM), China National Knowledge Internet (CNKI), and Embase by combining MeSH words with free words.. References to all included literature were screened to avoid omissions. The retrieval time was from the inception of the database to February 8, 2022. There were no language restrictions on literature searches, and the search strings used can be found in **Supplementary Table S1**.

### Study Selection and Quality Assessment

Titles and abstracts were screened separately by two researchers (Wang Y and Zhao J) for potential eligibility for inclusion. Any disagreements between researchers were resolved through consultation with the third researcher (Qin Y). Inclusion criteria: (1) the gut microbiota of patients with DKD was analyzed and its diversity or abundance was reported; (2) the observational case-control studies or cross-sectional studies design was used; (3) the adult population (18 years and older). Exclusion criteria: (1) only abstracts can be obtained and the authors cannot be contacted; (2) there is no control group or the control group is a non-healthy population; (3) combined with other metabolic diseases; (4) animal studies or studies of the microbiome outside the gut. Moreover, we used the Newcastle-Ottawa scale (NOS) to score the included literature (30).

### Data Extraction

Data were extracted independently by two researchers (Wang Y and Zhao J) and confirmed by the third researcher (Qin Y), using a unified extraction table including publication details, alpha diversity, beta diversity, differentially abundant microbial taxa, metabolites, and functional characteristics of gut microbiota. Alpha diversity provides a summary of microbial communities in a single sample and evaluates the richness and evenness of the sample. Beta diversity is a method of measuring inter-individual diversity, which assesses the similarity between communities and other analyzed sample.

### Data Processing and Meta-Integration

For alpha diversity, the RevMan5.3 software provided by the Cochrane collaboration network was used for Meta-merging. The minimum, mean (M), maximum, and standard deviation (SD) of alpha diversity indexes were extracted. If the median and quartile range in the original data were only provided, we used a web-based tool (http://www.math.hkbu.edu.hk/~tongt/papers/median2mean.html) to convert it to M and SD. If necessary, WebPlotdigitizer (V.4.4) was employed to extract digital data from the picture (31). The standardized mean difference (SMD) and 95% confidence interval (CI) of the above indexes between the DKD and controls were calculated. Two-sided P-values were statistically significant at less than 0.05. Heterogeneity is represented by *I* ^2^, and 0% means no statistical heterogeneity, *I* ^2^ *≤* 50% adopts a fixed-effects model, and *I* ^2^>50% adopts a random-effects model and analyzes the source of heterogeneity. The results were presented by forest plots and the publication bias by funnel plots.

Since the results of beta diversity and relative abundance of microbial taxa in the included studies were only qualitatively described (higher or lower in disease groups compared to controls) and no quantitative data were provided, a meta-analysis could not be performed. Therefore, we performed a semi-quantitative analysis; that is, the consistent findings from two or more studies are considered worth noting for future validation and may be relevant to the disease and as candidates for disease-specific responses. However, results reported only by one study were not analyzed because they were considered potential methodology or population-specific discrepancy.

## Results

### Search Results

**Figure 1:**
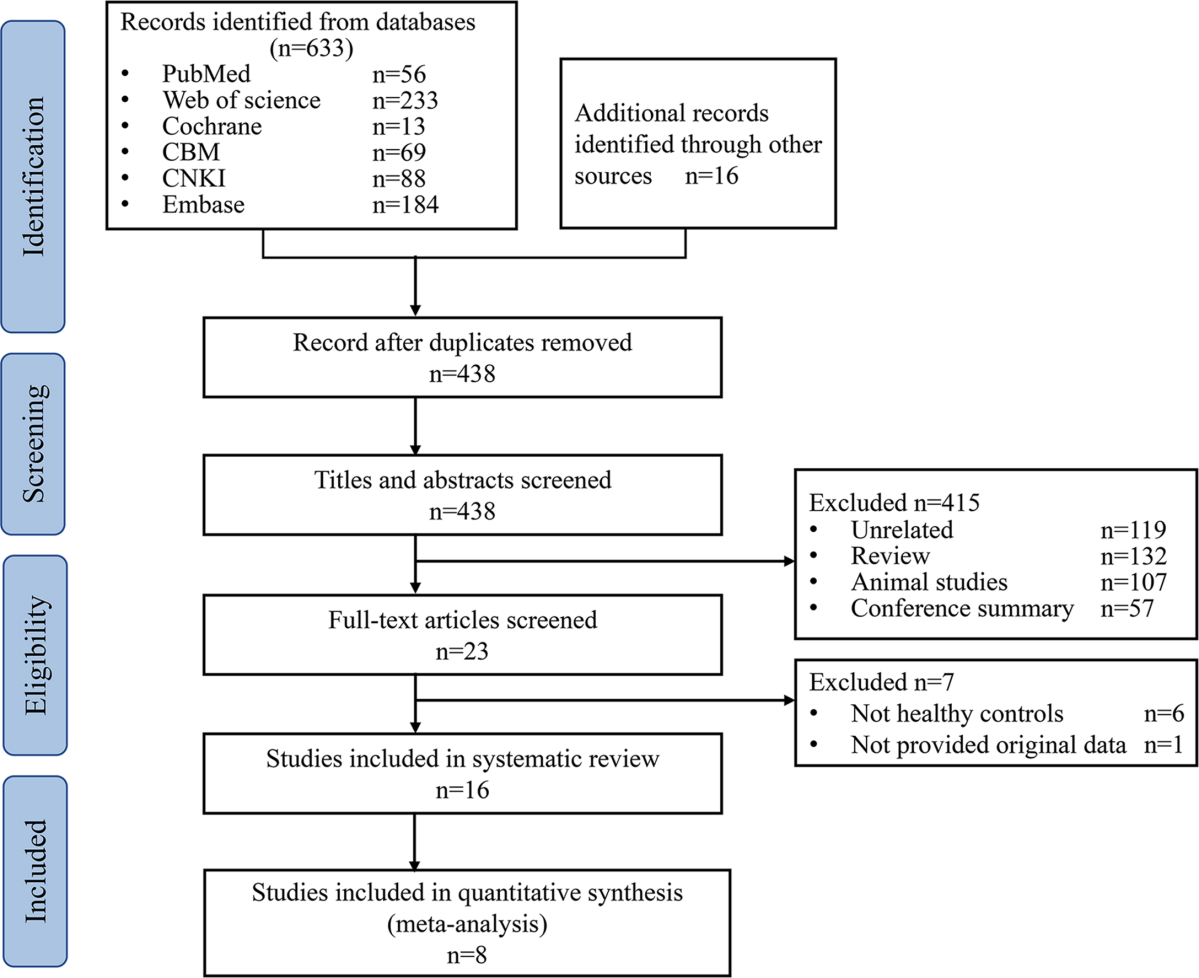
PRISMA flowcharts of study selection. CBM, Chinese Biomedical Databases. CNKI, China National Knowledge Internet.

A total of 649 studies were retrieved, 211duplicated studies were removed, 422 studies were removed according to inclusion and exclusion criteria, and 16 studies (17, 25–28, 32–42) were finally included (PRISMA flowcharts in **Figure 1**).

### Characteristics of Included Studies

The 16 studies included 578 patients with DKD and 444 HC, 12 (12/16) studies were conducted in Asia (China) and four (4/16) in Europe (Denmark, the United States, and Romania). Two (2/16) studies were diabetic nephropathy (DN) confirmed by renal biopsy, and the rest (14/16) were DKD diagnosed empirically or excluded clinically. Eleven (11/16) studies were analyzed by 16S rRNA gene amplification, three (3/16) by bacterial culture, one (1/16) by polymerase chain reaction-denaturing gradient gel electrophoresis (PCR-DEEG), and one (1/16) by shotgun metagenomics. Due to the differences in analysis methods of gut microbiota, the treatment methods of stool samples are also different. Samples were stored at -80°C in 11 (11/16) studies, -20°C in one (1/16) study, 4°C in one (1/16) study, and DNA was extracted within 24h in two (2/16) studies (**Table 1**).

**Table 1:** Characteristics of the included studies.

Study	Region-State	Object	Case (N, age)	Control (N, age)	Inclusion criteria of DKD	Analysis methods	Gender (male/female)	Sample storage
Sibei Tao et al. 2019 (33)	Sichuan, China	DN vs. T2D vs. HC vs. HH	DN 14, 52.93 ± 9.98 T2D 14, 53.29 ± 9.00	HC 14, 52.86 ± 9.91 HH 14, 44.29 ± 17.31	eGFR≧60mL/min/1.73 m^2^ and UACR≧30mg/g	16sRNA microbial profiling approach	DN, 9/5 T2D, 9/5 HC, 9/5 HH, 4/10	Stored at -80°C
Bao Xuguang et al. 2019 (26)	Guangzhou, China	DKD vs. T2D vs. HC	DKD 25, 63.7 ± 13.3 T2D 30, 63.7 ± 13.3	HC 30, 60.2 ± 9.7	Massive proteinuria with diabetes or DR with any stage of CKD or microalbuminuria in T1D with a course of more than 10 years	High-throughput sequencing technology	DKD, 16/9 T2D, 14/16 HC, 14/16	Stored at -80°C
Feng Chunnian et al. 2020 (27)	Sichuan, China	DKD vs. T2D vs. HC	DKD 57, 55.23 ± 11.21 T2D 68, 54.36 ± 11.12	HC 36, 54.36 ± 11.12	Not on dialysis	High-throughput sequencing technology	DKD, 34/23 T2D, 36/32 HC, 21/15	Stored at -80°C
Li Lei et al. 2021 (32)	Zhejiang, China	DKD vs. T2D vs. HC	DKD 20, 66.92 ± 9.27 T2D 20, 66.24 ± 10.24	HC 30, 67.41 ± 9.47	UAER >20μg/min	Bacterial culture	DKD, 11/9 T2D, 10/10 HC, 15/15	Stored at 4°C
Li Lei et al. 2020 (37)	Zhejiang, China	DKD vs. T2D vs. HC	DKD 25, 60∼96 T2D 21, 60∼96	HC 25, 60∼96	Massive proteinuria or DR with microalbuminuria	Bacterial culture	DKD, N/A T2D, N/A HC, N/A	Stored at -80°C
Lin Hao et al. 2020 (39)	Fujian, China	DKD vs. T2D vs. HC	DKD 68, 56.30 ± 4.26 T2D 72, 56.25 ± 4.32	HC 70, 55.24 ± 5.38	N/A	Bacterial culture	DKD, 40/28 T2D, 41/31 HC, 40/30	Stored at -80°C
Song Dandan et al. 2021 (40)	Inner Mongolia, China	DKD vs. T2D vs. HC	DKD 20, 58.2 ± 9.4 T2D 20, 54.1 ± 13.5	HC 20, 50.2 ± 12.6	eGFR: 22.4 ± 14.4mL/min/1.73 m^2^; SCr: 512.9 ± 364.6μmol/L	16S rRNA gene sequencing analysis	DKD, 12/8 T2D, 10/10 HC, 9/11	Stored at-80°C
Chun Huan et al. 2021 (41)	Zhejiang, China	DKD vs. T2D vs. HC	DKD 42, 69.06 ± 11.23 T2D 53, 68.47 ± 10.82	HC 42, 67.11 ± 9.26	UAER: 20~200μg/min; 24h UAER: 30~300mg/24h	16S rRNA gene sequencing analysis	DKD, 26/21 T2D, 31/22 HC, 24/18	Stored at-80°C
Sun Ya xian et al. 2016 (42)	Liaoning, China	DKD vs. T2D vs. HC	DKD 29, 59.17 ± 5.51 T2D 23, 55.87 ± 6.46	HC 20, 50.10 ± 5.19	ACR: 30~299mg/g	PCR-DEEG	DKD, 19/10 T2D, 11/12 HC, 5/15	Stored at-80°C
Li Ya et al. 2019 (36)	Guizhou, China	DKD vs. T2D vs. HC	DKD 10, N/A T2D 5, N/A	HC 5, N/A	ACR≧2.5 mg/mmol(male), ACR≧3.5 mg//mmol (famale), or 24h UALB ≧30 mg/24 h; and eGFR >15 mL/min/1.73 m^2^	16S rRNA gene amplicon sequencing	DKD N/A, T2D, N/A HC, N/A	Stored at -80°C
Xin Xiaohong et al. 2021 (25)	Shanxi, China	DN vs HC	DN 20, 55.1 ± 13.83	HC 20, 50.9 ± 9.49	SCr: 103 ± 17.72μmol/L	Metagenomic sequencing	DN, 10/10 HC, 10/10	N/A
Xi Du et al. 2021 (28)	Tianjin, China	DKD vs. HC	DKD 43, 60.86 ± 5.69	HC 37, 61.78 ± 6.40	Stage 3 or 4 of DKD	16S ribosomal DNA gene sequencing	DKD, 32/11 HC, 25/12	Stored at -80°C
Mohammed A. I. Al-Obaide et al. 2017 (34)	the United States of America	DKD vs. HC	DKD 20, 64.4 ± 2.3	HC 20, 54.3 ± 3.2	GFR < 30 mL/min/1.72 m^2^ and not on dialysis	16S rRNA gene sequencing analysis	DKD, N/A HC, N/A	DNA extraction within 24 h
Maria V. Salguero et al. 2019 (17)	the United States of America	DKD vs. HC	DKD 20, 62.8 ± 3.6	HC 20, 58.5 ± 4.1	Stages 4 and 5 of CKD, not on dialysis; GFR < 30 ml/min/1.72 m^2^	16S rRNA gene sequencing analysis	DKD, 9/11 HC, 11/9	DNA extraction within 24 h
Gratiela P. Gradisteanu et al. 2019 (38)	Romania	DKD vs. HC	DKD 9, N/A	HC 5, N/A	N/A	16 rDNA qRT-PCR	DKD, N/A HC, N/A	Freezing at -20°C
Signe A. Winther et al. 2020 (35)	Denmark	DKD (T1D: Nor, Mic, Mac) vs. HC	T1D 161, 60 ± 10	HC 50, 59 ± 13	UACR≧30mg/g	16S rRNA gene amplicon sequencing	T1D, 94/67 HC, 28/22	Stored at -80°C

DKD, diabetic kidney disease; HC, healthy controls; T2D, type 2 diabetes; HH, household contacts; DN, diabetic nephropathy confirmed by renal biopsy; T1D, type 1 diabetes; Nor, normoalbuminuria; Mic, microalbuminuria; Mac, macroalbuminuria; PCR, polymerase chain reaction; DEEG, denaturing gradient gel electrophoresis; DR, diabetic retinopathy; CKD, chronic kidney disease; eGFR, estimated glomerular filtration rate; UAER, urinary albumin excretion rates; UACR, urine albumin creatinine ratio; ACR, urine albumin-to-creatinine ratio; SCr, serum creatinine; GFR, the glomerular filtration rate; N/A, not available.

### Quality of Included Studies

The included studies were evaluated by Newcastle-Ottawa Scale (30), and five (5/16) studies were ranked with 8*, seven (7/16) with 7*, and four (4/16) with 6*, indicating that the quality of the selected studies was generally high (**Supplementary Table S2**).

### Alpha Diversity

**Figure 2:**
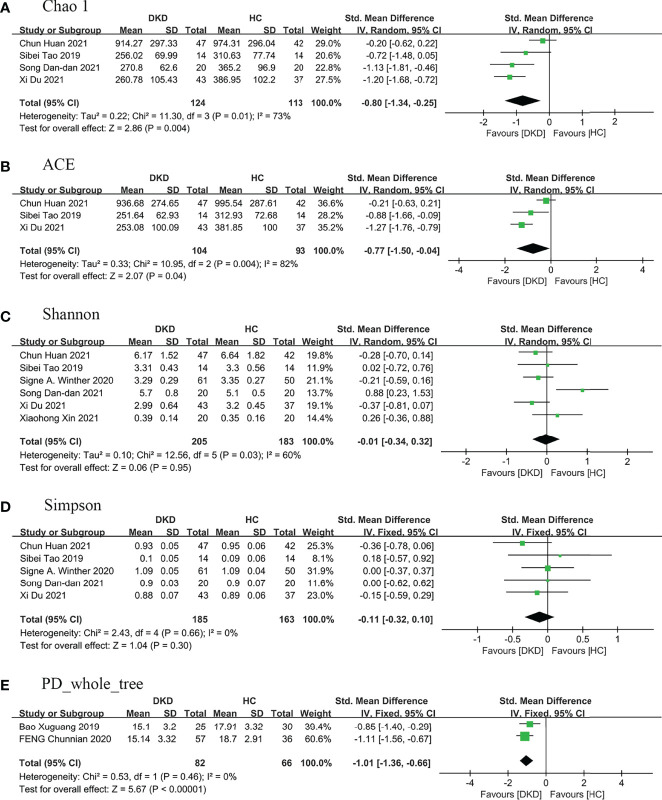
Forest plots of alpha diversity in the gut microbiota of patients with DKD compared with HC. **(A)** Chao1 index, **(B)** ACE index, **(C)** Shannon index, **(D)** Simpson index, **(E)** PD_whole_tree index. A and B represent richness, while C, D, and E represent evenness. PD_whole_tree, phylogenetic diversity whole tree. DKD, diabetic kidney diseases; HC, healthy controls.

Nine (9/16) studies (25–28, 33, 35, 40–42) analyzed alpha diversity between DKD and HC, mainly related to two factors (31): one is richness, the number of species; second is diversity, the evenness of individual distribution in a community. The indexes of community richness mainly include Chao1, ACE, Observed species, and Sob. The indexes of community diversity, including Shannon, Simpson, Fisher, and phylogenetic diversity whole tree (PD _whole _tree) (**Supplementary Table S3**). A meta-analysis was performed on the alpha diversity indexes reported in two or more studies (**Figure 2**), and publication bias assessments were presented in **Supplementary Figure S1**.

Regarding richness, four studies (28, 33, 40, 41) provided Chao1 index for quantitative consolidation (SMD = -0.80, 95% CI -1.34 to -0.25, *p* = 0.004, *I* ^2^ = 73%) and three studies (28, 33, 41) reported ACE index (SMD = -0.77, 95% CI -1.50 to -0.04, *p* = 0.04, *I* ^2^ = 82%). The number of species in the gut microbiota in DKD decreased significantly.

Regarding diversity, six studies (25, 28, 33, 35, 40, 41) reported the Shannon index for quantitative consolidation (SMD = -0.01, 95% CI -0.34 to 0.32, *p* = 0.95, *I* ^2^ = 60%), five studies (28, 33, 35, 40, 41) provided the Simpson index (SMD = -0.11, 95% CI -0.32 to 0.10, *p* = 0.30, *I* ^2^ = 0%), the results suggested that the species diversity of gut microbiota decreased in DKD, but there was no statistical significance. Two studies (26, 27) provided PD _whole _tree (SMD = -1.01, 95% CI -1.36 to -0.66, *p <*0.00001, *I* ^2^ = 0%), and suggested phylogenetic diversity reduced and no heterogeneity in DKD.

We performed sensitivity analyses on indicators with greater heterogeneity. After removing the article of Chun Huan et al. (41), the heterogeneity of Chao1 and ACE decreased to 0%, the bacterial richness of patients with DKD decreased, and the statistical difference was more significant (*I* ^2^ = 0%, *p <*0.00001). The subjects of this study (41) are mainly elderly patients with DKD compared with that of other studies (28, 33, 40), and the age difference of patients may be the main source of heterogeneity. After excluding the study of Song Dan-dan et al. (40), the heterogeneity of the Shannon index decreased to 0% (*I* ^2^ = 0%, *p* = 0.08). Compared with other studies (25, 28, 33, 35, 41), the subjects of the study (40) were mainly patients with an advanced renal function who did not enter dialysis, and the stage of renal function may be the main source of heterogeneity.

### Beta Diversity

Eight (8/16) studies (25–28, 33, 35, 40, 42) reported the beta diversity of gut microbiota between patients with DKD and HC, six (25–27, 33, 35, 40) of which (6/8) adopted principal coordinate analysis (PCoA), one (1/8) study (42) used principal component analysis (PCA), and one (1/8) study (28) used PCoA, PCA, and nonmetric multidimensional scale (NMD). The results showed that the beta diversity of DKD was significantly different from that of HC (**Supplementary Table S3**).

### Differentially Abundant Microbial Taxa

Currently, the review identified 16 (16/16) studies (17, 25–28, 32–42) that compared the composition of gut microbiota in patients with DKD and HC (**Supplementary Table S4**).

Six (6/16) studies (17, 26–28, 33, 40, 41) described the distinct taxa at the phylum level, two (2/6) of which found that the relative abundance of *Proteobacteria (* 17, 33), *Actinobacteria* (17, 33), and *Bacteroidetes (* 27, 41) in patients with DKD was higher than that in HC. Five (5/6) studies found that the relative abundance of *Firmicutes (* 26, 27, 33, 40, 41) in patients with DKD was lower.

Two (2/16) studies (28, 35) reported the distinct taxa at the class level, and no consistent changes were found.

Three (3/16) studies (28, 35, 40) explored the relative distinct taxa at the order level and found that the abundance of *Selenomonadales* was increased in DKD in T2D (28) and decreased in DKD in type 1 diabetes (35) compared with HC.

Five (5/16) studies (17, 26, 28, 33, 38) showed the distinct taxa at the family level and found that the relative abundance of *Enterobacteriaceae (* 17, 26, 38), *Coriobacteriaceae (* 26, 33), and *Veillonellaceae (* 26, 28) were enriched in DKD compared with HC, while *Lachnospiraceae (* 26, 28) was depleted.

Fifteen (15/16) studies (17, 25–28, 32–40, 42) reported the distinct taxa at the genus level, and found that the enrichment of *Citrobacter (* 17, 25, 34), *Escherichia (* 17, 25, 34), *Klebsiella (* 17, 25, 34), *Enterococcus (* 26, 34), *E. coli (* 32, 37) (bacterial culture), *Akkermansia (* 17, 25), *Sutterella (* 17, 28), and *Acinetobacter (* 17, 34) in DKD compared with HC, whereas the depletion of *Bifidobacterium (* 27, 34), *Bifidobacteria (* 37, 39) (bacterial culture), *Prevotella (* 25, 42), and *Roseburia (* 26, 28) in DKD. In addition, the abundance variations of *Lactobacillus* (increased in three studies (26, 28, 34) and decreased in two studies (39, 42)), *Coprococcus* (increased in three studies (27, 36, 39) and decreased in one study (26)), and *Proteus* (increased in two studies (17, 34) and decreased in one study (39)) are controversial, and *Lachnoclostridium (* 25, 28), *Rothia (* 25, 27),and *Lachnospira (* 26, 27) were found to have contrary conclusions in two studies, which need to be further verified in future studies.

### The Phylogenetic Profile of the Differentially Abundant Taxa

**Figure 3:**
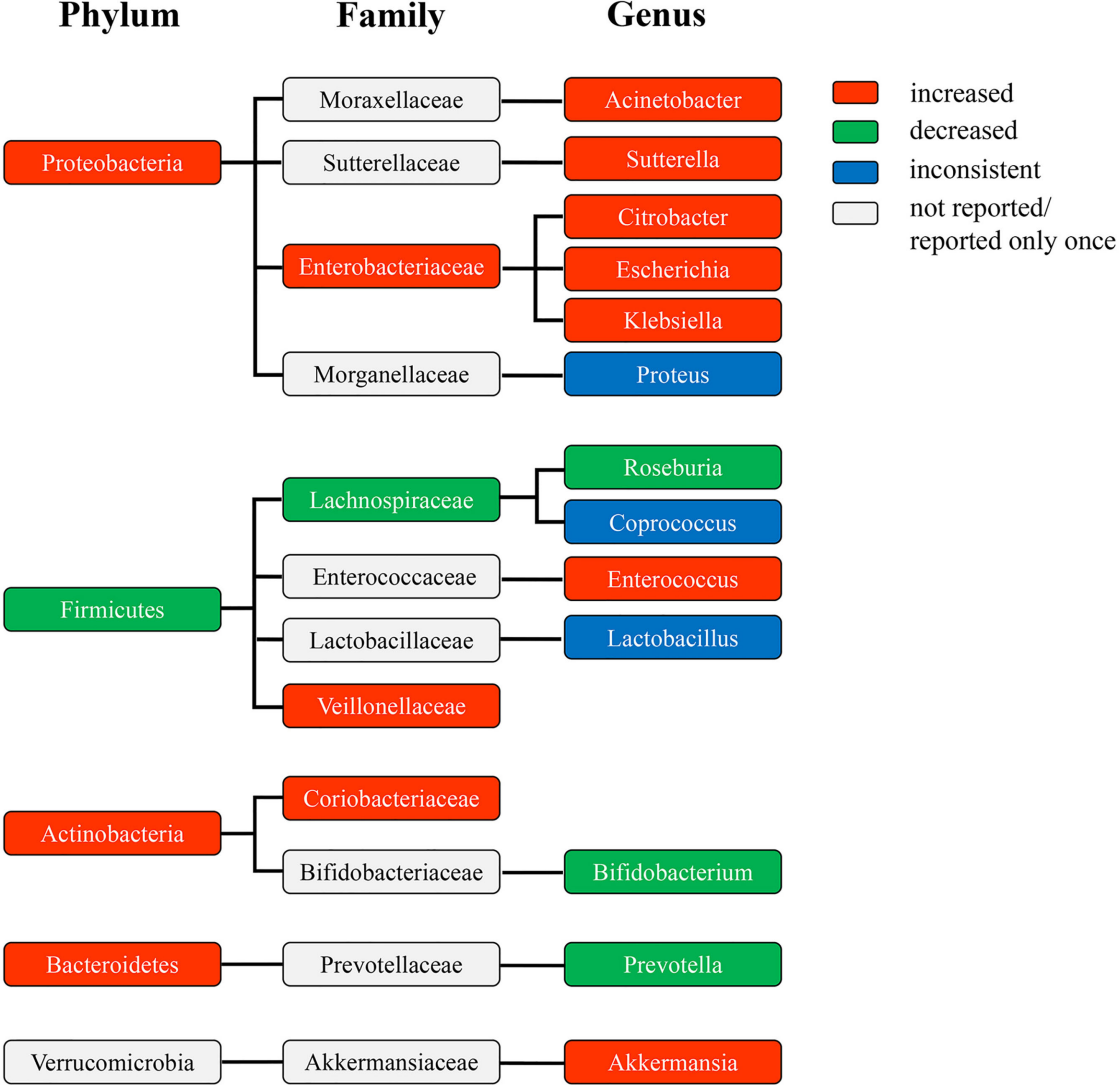
The phylogenetic profile of differentially abundant taxa at the phylum, family, and genus level of patients with DKD compared to HC. The colors indicate different bacterial variations at the phylum, family, and genus levels. Red, abundance increased. Green, abundance decreased. Blue, abundance inconsistent. Gray, abundance not reported or reported only once. DKD, diabetic kidney diseases; HC, healthy controls.

The phylogenetic profile of the differentially abundant taxa in patients with DKD at the phylum, family, and genus level showed that the amplification of genus *Citrobacter*, *Escherichia*, and *Klebsiella* may make a great contribution to the increase of phylum *Proteobacteria*, and the depletion of genus *Roseburia* significantly contributed to the decrease of phylum *Firmicutes* (**Figure 3**). Specifically, the abundance of genus *Acinetobacter*, *Sutterella*, *Citrobacter*, *Escherichia*, and *Klebsiella* increased in the enriched phylum *Proteobacteria*, while only genus *Citrobacter*, *Escherichia*, and *Klebsiella* were consistent with the increased abundance with their corresponding family-level (*Enterobacteriaceae*), indicating that they varied dramatically in abundance and may contribute most to the expansion of phylum *Proteobacteria*. Among the decreased phylum *Firmicutes*, the depletion of genus *Roseburia* may contribute to the decrease of family *Lachnospiraceae*, suggesting that the abundance of genus *Roseburia* reduced significantly and may contribute greatly to the depletion of phylum *Firmicutes*. However, the increase of genus *Enterococcus* may not promote the change of family *Enterococcaceae*, which thus exert less influence on the abundance of phylum *Firmicutes.* The decrease of the abundance of genus *Bifidobacterium* may not cause a significant variation in the abundance of family *Bifidobacteriaceae*, and was contrary to the variation enrichment of the abundance of phylum *Actinobacteria*. The enrichment of family *Coriobacteriaceae* may promote the increase of phylum *Actinobacteria*, but no significantly differential taxa were found at the genus level. The alteration of the abundance of genus *Prevotella and Akkermansia* did not cause the consistent changes in the abundance of their corresponding family and phylum taxa, respectively, suggesting their little influence on the change of gut microbiota in patients with DKD.

### Inflammatory Indicators, Intestinal Barrier Dysfunction Markers, and Gut Microbiota Metabolites

Five (5/15) studies (17, 26, 27, 34, 37) reported inflammatory markers and intestinal barrier function between DKD and HC, and found that inflammatory indicators in patients with DKD were higher than those in HC (e.g.: IL-6 (17, 26, 27, 34, 37), CRP [increased in four studies (17, 26, 27, 37), and no statistical difference in one study (34)], TNFα (17, 34, 37), IL-8 (37), LPS [increased in one study (17) and no statistical difference in the other study (34)]. The levels of intestinal permeability index (zonulin) and endothelial dysfunction marker (ET-1) in DKD were higher than those in controls (34) (**Supplementary Table S5**).

Two (2/16) studies (34, 35) examined gut microbiota metabolites between DKD and HC. Mohammed A. I. Al-Obaided et al. demonstrated that the serum levels of trimethylamine-N-oxide (TMAO) in patients with DKD were significantly higher than in HC (34). Signe A. Winther et al. found that compared with HC, patients with T1D with macroalbuminuria had lower concentrations of tryptophan and higher concentrations of L-citrulline (35).

### Functional Characteristics of Gut Microbiota

Only one (1/16) study (25) used the KEGG method to evaluate the functional characteristics of gut microbiota in patients with DKD and HC, and found overexpression of the tyrosine metabolic pathway and low expression of a short-chain fatty acid metabolic pathway in DKD (**Supplementary Table S6**).

## Discussion

To our knowledge, this is the first meta-analysis to systematically evaluate gut dysbiosis in patients with DKD to identify specific microbial taxa that may contribute to the onset or progression of DKD. There is consistent evidence that the composition of the gut microbiota is specifically altered in DKD, in which phylogenetic analysis of the differentially abundant taxa showed that the expansion of genus *Escherichia*, *Citrobacter*, and *Klebsiella*, and the depletion of *Roseburia* may make a great contribution to gut dysbiosis in DKD, which may provide bacterial targets for preventing or treating DKD by reestablishing the homeostasis of gut microbiota and be worthy of verification in future studies.

Lower alpha diversity is considered detrimental to the host (43) and has been observed in a range of other clinical conditions, including obesity, type 1 and 2 diabetes, and inflammatory bowel disease (IBD) (44). However, regarding alpha diversity (within sample), our meta-analysis demonstrated no significant association with diversity indexes (evenness) and richness, suggesting that while richness is compromised, evenness is overall preserved. While the diversity of DKD is not significantly different from that of T2D (26, 27), suggesting that alpha diversity is not a good discriminator of disease progression. Regarding beta diversity (between samples), the composition of gut microbiota in patients with DKD and HC was significantly different. Together, the above findings preliminarily revealed that the profile of gut microbiota was altered in patients with DKD.

*Escherichia and Acinetobacter* are TMAO-producing bacteria (34, 45) and are enriched in DKD compared with HC. TMAO was verified extensively to induce vascular inflammatory, endothelial dysfunction, and foam cell formation by activating nuclear factor-kappaB (NF-κB) and mitogen-activated protein kinases (MAPK)-related pathways, leading to atherosclerosis (46), renal damage, and fibrosis (47), and it is considered to be an independent gut microbiota-derived risk factor for cardiovascular and renal disease. Meanwhile, elevated TMAO levels in plasma were found to be positively associated with the prevalence of T2D (48) and were predictive of mortality, cardiovascular disease, and renal outcomes in T1D and T2D (49, 50), which was consistent with the enrichment of *Escherichia* in T1D and T2D (51, 52). TMAO binding and activating PERK may be one of the potential mechanisms of diabetes (53). *Citrobacter* was increased in DKD compared with HC, while no significant enrichment was found in T1D and T2D. *Citrobacter* infection may lead to loss of intestinal barrier integrity and mucosal damage by disrupting the mitochondrial structure and oxidative respiratory chain function in intestinal epithelial cells (54), accelerating insulitis *via* activation of diabetogenic CD8^+^ T cells (55) and allowing pathogens and their derived toxins to enter the bloodstream, resulting in chronic inflammation and uremia (56), which may contribute to the occurrence and development of DKD. Besides, *Escherichia* may also enhance intestinal infiltration by penetrating the intestinal epithelial barrier (57), leading to the escape of pathogenic and commensal bacteria from the intestinal lumen to activate the systemic immune system.

Chronic inflammation plays an important role in the occurrence and development of diabetes and DKD (58). *Escherichia*, *Klebsiella*, and *Sutterella* are increased in DKD, T1D (52, 59, 60), and pre-and untreated T2D (51, 61), which can effectively increase the circulating LPS levels (56, 62, 63). LPS can bind to Toll-like receptor (TLR) 4 resulting in inflammatory responses, cytokine production, and chemokine-mediated recruitment of inflammatory cells (64), which may damage cellular insulin receptors (65) and pancreatic β-cell (66), leading to insulin resistance (67) and insulin deficiency, and promoting the occurrence of diabetes. High serum LPS activity predicts the progression of T1D to DKD. Treatment of podocytes with LPS decreased the expression of 3-phosphoinositide-dependent kinase-1 (PDK1), stimulated the pro-apoptotic p38MAPK pathway, and induced apoptosis, which contributed to the occurrence of DKD, while the immunomodulator 4,5-Dihydro-3-phenyl-5-isoxazoleacetic acid (GIT27), which inhibits the TLR signal pathway, can prevent this pathological change (68). In addition, Yoshihiko Sawa et al. found that TLR combined with LPS to produce cytokines, such as TNFα and IL-6, transforming growth factor-β (TGF-β), which promotes the increase of proteinuria and the accumulation of type I collagen in the glomeruli in type 1 and type 2 diabetic mice (69). The above evidence suggests that reducing plasma LPS concentrations by altering the gut microbiota may be an effective strategy to control the progression of diabetes and DKD. Interestingly, consistent with the proinflammatory properties of the above differentially abundant bacteria, we found that LPS and other inflammatory markers (e.g., TNFα, CRP, IL-6, etc.) were elevated in the serum of patients with DKD (**Supplementary Table S5**).

*Akkermansia* was reported to be negatively associated with untreated T2D (12), and animal studies found that genus *Akkermansia* mediates the negative regulation of glucose metabolism by interferon gamma (IFNγ) (70). Supplementation with *Akkermansia* can improve insulin sensitivity, improve blood lipids, and reduce LPS (71, 72). Unexpectedly, our study found that genus *Akkermansia* is enriched in DKD, which may be related to the use of hypoglycemic drugs in patients with DKD. Animal and human studies have shown that metformin can increase the abundance of *Akkermansia* in the gut microbiota (73, 74). Therefore, longitudinal studies evaluating the effects of drugs on the gut microbiota are necessary to select the optimal treatment options.

*Roseburia* is a butyrate-producing bacteria and is depleted in DKD, T1D (75), and T2D (76). Butyrate, a kind of short-chain fatty acids, can stimulate the colonic L cells to secrete a large amount of GLP-1 and a minor amount of PYY by activating the colonic free fatty acid receptors FFAR2 (GPR43) and FFAR3 (GPR41), and GLP-1 and PYY have the potential anti-diabetes and anti-obesity effects (77). Studies have shown that butyrate combined with GLP-1 can promote differentiation of pancreatic progenitor cells into insulin-producing cells, inducing the proliferation of insulin-producing cells, promoting the synthesis and release of insulin (78), improving blood glucose tolerance, insulin resistance, dyslipidiasis, and inflammation, and reducing body fat in diabetic patients (79). PYY promotes pancreatic β-cell proliferation and reduces apoptosis by activating hypothalamic neuropeptide Y1 receptors (NPYR1) (80, 81), and increases intestinal transit rate and satiety by activating NPYR2, which have obvious benefits for diabetes and obesity (81). In addition, butyrate has a protective effect on DKD by promoting the expression of GPR43 and inhibiting the activation of NF-kB (82). These evidences suggest that *Roseburia* and its metabolites may be potential therapeutic targets for diabetes and DKD.

**Figure 4:**
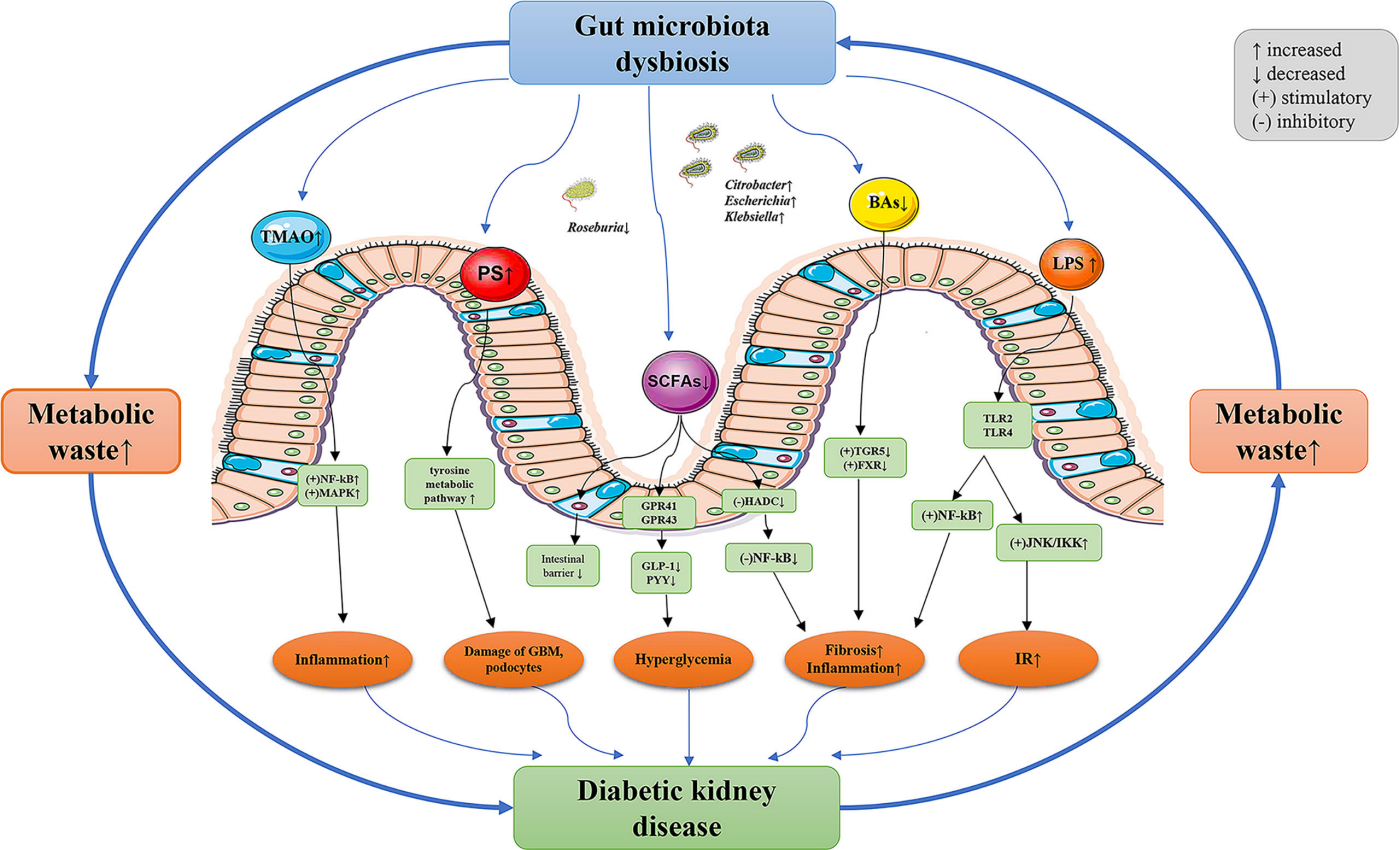
The potential mechanism between gut dysbiosis and DKD. The decreased glomerular filtration rate of DKD patients leads to the accumulation of metabolic waste, which enters the intestinal lumen through the intestinal wall and results in gut dysbiosis. The composition and function of gut microbiota in patients with DKD significantly varied, resulting in the destruction of intestinal epithelial barrier function. Gut microbiota metabolites enter the blood and further aggravate the progression of DKD through a variety of pathways. TMAO, trimethylamine-N-oxide; PS, phenyl sulfate; SCFAs, short-chain fatty acids; BAs, bile acids; LPS, lipopolysaccharide; NF- κB, nuclear factor kappa-B; MAPK, mitogen-activated protein kinases; G-protein-coupled receptors (GPRs), such as GPR41 and GPR43; GLP-1, glucagon-like peptide-1; PYY, peptide YY; HADC, histone deacetylases; TGR5, Takeda G-protein receptor 5; FXR, farnesoid X receptor; TLR, toll-like receptors; JNK, c-Jun N-terminal kinase; IKK, IkappaB kinase; IR, insulin resistance; GBM, glomerular basement membrane; DKD, diabetic kidney diseases.

Although only one study analyzed the functional characteristics of the gut microbiota between DKD and HC, the results were in agreement with the gut microbiota, further supporting the potential role of gut microbiota in the regulation of DKD. The KEGG analysis showed that the tyrosine metabolic pathway was overexpressed and the short-chain fatty acid metabolic pathway was under-expressed in DKD. Kikuchi et al. (83) confirmed in a variety of animal models that phenyl sulfate (PS) produced by tyrosine metabolism promoted the production of proteinuria by leading to damage of the glomerular basement membrane barrier and podocyte, inhibiting the key enzyme in its metabolic pathway (tyrosine phenol lyase, TPL) can reverse the above-mentioned renal pathological changes. They also found that the enrichment of family *Coriobacteriaceae*- genus *Adlercreutzia* was positively correlated with the concentration of PS in serum. Our study concluded a consistent finding on the enrichment of family *Coriobacteriaceae*, albeit no differential taxa at the genus level have been found, which needs to be validated in future studies. In addition, the low expression of short-chain fatty acid metabolic pathways is consistent with the reduction of short-chain fatty acid-producing bacteria. In conclusion, the analysis of the metabolic pathways of gut microbiota further supports the characteristics of gut dysbiosis in DKD, and the intervention of the metabolic pathways of specific microbiota in DKD may provide a new strategy for the prevention and treatment of DKD. The potential mechanism between gut dysbiosis and DKD was postulated and is shown in **Figure 4**.

Some limitations should be noted. First, the paucity of quantitative data on all parameters of gut microbiota impeded us from performing a comprehensive meta-analysis, which may influence the strength of evidence. Second, the crucial microbial taxa closely related to the onset or progression of DKD were not fully determined due to the feature of observational studies included in our study. Third, due to the incomplete data reporting, we were unable to conduct a subgroup analysis to assess the effects of some factors such as eating habits, living environment, obesity condition, and drugs on the changes of gut microbiota in patients with DKD. Therefore, future endeavors will seek to discover the cause and effect between gut microbiota and DKD and the underlying mechanism.

## Conclusions

The gut microbiota of patients with DKD may possess specific characteristics. Compared to HC, the richness of gut microbiota in patients with DKD was decreased, while the diversity indexes were overall preserved, and the beta diversity was significantly distinct. Alterations of genus *Escherichia*, *Citrobacter*, *Klebsiella*, and *Roseburia* may contribute most to the abundance variation of their corresponding family and phylum taxa, as well as bacterial diversity and composition. These microbial taxa may be closely related to DKD and serve as promising targets for the management of DKD, which warrants further exploration.

## Data Availability Statement

The original contributions presented in the study are included in the article/**Supplementary Material**. Further inquiries can be directed to the corresponding author.

## Author Contributions

YW, JZ, and SS wrote the manuscript and researched the data. YQ researched data, contributed to discussion, and reviewed the manuscript. ZY, YZ, and XN contributed to the discussion and reviewed the manuscript. All authors read and approved the final version of the manuscript, and take responsibility for the integrity of the data and the accuracy of the data analysis.

## Funding

National Natural Science Foundation of China grants (Reference number: 82170722 and 81870470), Key project of Shaanxi province (Reference number: 2017ZDXM-SF-045), and Clinical research project of Air Force Military Medical University (Reference number: 202ILC2205) supported this study.

## Conflict of Interest

The authors declare that the research was conducted in the absence of any commercial or financial relationships that could be construed as a potential conflict of interest.

## Publisher’s Note

All claims expressed in this article are solely those of the authors and do not necessarily represent those of their affiliated organizations, or those of the publisher, the editors and the reviewers. Any product that may be evaluated in this article, or claim that may be made by its manufacturer, is not guaranteed or endorsed by the publisher.

## References

[1] Federation ID . Idf Diabetes Atlas— (2021). Available at: http://www.diabetesatlas.org/. 35914061

[2] Afkarian M Zelnick LR Hall YN Heagerty PJ Tuttle K Weiss NS . Clinical Manifestations of Kidney Disease Among Us Adults With Diabetes, 1988-2014. Jama (2016) 316(6):602–10. doi: 10.1001/jama.2016.10924 PMC5444809 27532915

[3] de Boer IH . Kidney Disease and Related Findings in the Diabetes Control and Complications Trial/Epidemiology of Diabetes Interventions and Complications Study. Diabetes Care (2014) 37(1):24–30. doi: 10.2337/dc13-2113 24356594 PMC3867994

[4] Johansen KL Chertow GM Foley RN Gilbertson DT Herzog CA Ishani A . Us Renal Data System 2020 Annual Data Report: Epidemiology of Kidney Disease in the United States. Am J Kidney Dis (2021) 77(4 Suppl 1):A7–a8. doi: 10.1053/j.ajkd.2021.01.002 33752804 PMC8148988

[5] Sagoo MK Gnudi L . Diabetic Nephropathy: An Overview. Methods Mol Biol (Clifton NJ) (2020) 2067:3–7. doi: 10.1007/978-1-4939-9841-8_1 31701441

[6] Alicic RZ Rooney MT Tuttle KR . Diabetic Kidney Disease: Challenges, Progress, and Possibilities. Clin J Am Soc Nephrol CJASN (2017) 12(12):2032–45. doi: 10.2215/cjn.11491116 PMC5718284 28522654

[7] Samsu N . Diabetic Nephropathy: Challenges in Pathogenesis, Diagnosis, and Treatment. BioMed Res Int (2021) 2021:1497449. doi: 10.1155/2021/1497449 34307650 PMC8285185

[8] Gilbert JA Blaser MJ Caporaso JG Jansson JK Lynch SV Knight R . Current Understanding of the Human Microbiome. Nat Med (2018) 24(4):392–400. doi: 10.1038/nm.4517 29634682 PMC7043356

[9] Kostic AD Gevers D Siljander H Vatanen T Hyötyläinen T Hämäläinen AM . The Dynamics of the Human Infant Gut Microbiome in Development and in Progression Toward Type 1 Diabetes. Cell Host Microbe (2015) 17(2):260–73. doi: 10.1016/j.chom.2015.01.001 PMC4689191 25662751

[10] Bakir-Gungor B Bulut O Jabeer A Nalbantoglu OU Yousef M . Discovering Potential Taxonomic Biomarkers of Type 2 Diabetes From Human Gut Microbiota *Via* Different Feature Selection Methods. Front Microbiol (2021) 12:628426. doi: 10.3389/fmicb.2021.628426 34512559 PMC8424122

[11] Vals-Delgado C Alcala-Diaz JF Roncero-Ramos I Leon-Acuña A Molina-Abril H Gutierrez-Mariscal FM . A Microbiota-Based Predictive Model for Type 2 Diabetes Remission Induced by Dietary Intervention: From the Cordioprev Study. Clin Trans Med (2021) 11(4):e326. doi: 10.1002/ctm2.326 PMC8023646 33931973

[12] Gurung M Li Z You H Rodrigues R Jump DB Morgun A . Role of Gut Microbiota in Type 2 Diabetes Pathophysiology. EBioMedicine (2020) 51:102590. doi: 10.1016/j.ebiom.2019.11.051 31901868 PMC6948163

[13] Zheng X Chen T Jiang R Zhao A Wu Q Kuang J . Hyocholic Acid Species Improve Glucose Homeostasis Through a Distinct Tgr5 and Fxr Signaling Mechanism. Cell Metab (2021) 33(4):791–803.e7. doi: 10.1016/j.cmet.2020.11.017 33338411

[14] Degirolamo C Rainaldi S Bovenga F Murzilli S Moschetta A . Microbiota Modification With Probiotics Induces Hepatic Bile Acid Synthesis *Via* Downregulation of the Fxr-Fgf15 Axis in Mice. Cell Rep (2014) 7(1):12–8. doi: 10.1016/j.celrep.2014.02.032 24656817

[15] Wang XX Wang D Luo Y Myakala K Dobrinskikh E Rosenberg AZ . Fxr/Tgr5 Dual Agonist Prevents Progression of Nephropathy in Diabetes and Obesity. J Am Soc Nephrol JASN (2018) 29(1):118–37. doi: 10.1681/asn.2017020222 PMC5748904 29089371

[16] Zhao L Zhang F Ding X Wu G Lam YY Wang X . Gut Bacteria Selectively Promoted by Dietary Fibers Alleviate Type 2 Diabetes. Sci (New York NY) (2018) 359(6380):1151–6. doi: 10.1126/science.aao5774 29590046

[17] Salguero MV Al-Obaide MAI Singh R Siepmann T Vasylyeva TL . Dysbiosis of Gram-Negative Gut Microbiota and the Associated Serum Lipopolysaccharide Exacerbates Inflammation in Type 2 Diabetic Patients With Chronic Kidney Disease. Exp Ther Med (2019) 18(5):3461–9. doi: 10.3892/etm.2019.7943 PMC6777309 31602221

[18] Ling LW Yongen C Vaziri ND . The Consequences of Altered Microbiota in Immune-Related Chronic Kidney Disease. Nephrol Dialysis Transplant (2020) 36(10):1791–8. doi: 10.1093/ndt/gfaa087 PMC8633451 32437554

[19] Lu CC Hu ZB Wang R Hong ZH Lu J Chen PP . Gut Microbiota Dysbiosis-Induced Activation of the Intrarenal Renin-Angiotensin System Is Involved in Kidney Injuries in Rat Diabetic Nephropathy. Acta Pharmacol Sin (2020) 41(8):1111–8. doi: 10.1038/s41401-019-0326-5 PMC7471476 32203081

[20] Bo HZ Jian L Pei CP Chen LC Xiu ZJ Qi LX . Dysbiosis of Intestinal Microbiota Mediates Tubulointerstitial Injury in Diabetic Nephropathy *Via* the Disruption of Cholesterol Homeostasis. Theranostics (2020) 10(6):2803–16. doi: 10.7150/thno.40571 PMC7052905 32194836

[21] Mafi A Namazi G Soleimani A Bahmani F Aghadavod E Asemi Z . Metabolic and Genetic Response to Probiotics Supplementation in Patients With Diabetic Nephropathy: A Randomized, Double- Blind, Placebo- Controlled Trial. Food Funct (2018) 9(9):4763–70. doi: 10.1039/c8fo00888d 30113051

[22] Manaer T Yu L Zhang Y Xiao X-J Nabi X-H . Anti-Diabetic Effects of Shubat in Type 2 Diabetic Rats Induced by Combination of High-Glucose-Fat Diet and Low-Dose Streptozotocin. J Ethnopharmacol (2015) 169:269–74. doi: 10.1016/j.jep.2015.04.032 25922265

[23] Koshida T Gohda T Kishida C Sakuma H Adachi E Masanori I . Pos-359 Potential Effect of Broad-Spectrum Antibiotic Therapy on Progression of Diabetic Kidney Disease in Mice. Kidney Int Rep (2021) 6(4):S156. doi: 10.1016/j.ekir.2021.03.376

[24] Lu J Chen PP Zhang JX Li XQ Wang GH Yuan BY . Gpr43 Deficiency Protects Against Podocyte Insulin Resistance in Diabetic Nephropathy Through the Restoration of Ampk Alpha Activity. Theranostics (2021) 11(10):4728–42. doi: 10.7150/thno.56598 PMC7978296 33754024

[25] Xin X . The Analysis of Intestinal Microbiota Characteristics and Potential Biomarkers in Patients With Diabetic Nephropathy [Master]. Shanxi: Shanxi Medical University (2021).

[26] Bao X Wang Z He Y Wang S Li Z Li P . Patterns of Intestinal Microbiome Imbalance in Patients With Type 2 Diabetes Mellitus and Diabetes Kidney Disease. Chin J Lab Med (2019) 42(6):469–78. doi: 10.3760/cma.j.issn.1009-9158.2019.06.014

[27] Feng C Zeng L Wang S Zhou H Luo X . Analysis of Microinflammation and Intestinal Microbial Diversity in Patients With Type 2 Diabetes Mellitus and Diabetes Kidney Disease. Chin J Microecol (2020) 32(11):1273–8. doi: 10.13381/j.cnki.cjm.202011006

[28] Du X Liu J Xue Y Kong X Lv C Li Z . Alteration of Gut Microbial Profile in Patients With Diabetic Nephropathy. Endocrine (2021) 73(1):71–84. doi: 10.1007/s12020-021-02721-1 33905112

[29] Hutton B Salanti G Caldwell DM Chaimani A Schmid CH Cameron C . The Prisma Extension Statement for Reporting of Systematic Reviews Incorporating Network Meta-Analyses of Health Care Interventions: Checklist and Explanations. Ann Intern Med (2015) 162(11):777–84. doi: 10.7326/m14-2385 26030634

[30] Stang A . Critical Evaluation of the Newcastle-Ottawa Scale for the Assessment of the Quality of Nonrandomized Studies in Meta-Analyses. Eur J Epidemiol (2010) 25(9):603–5. doi: 10.1007/s10654-010-9491-z 20652370

[31] Drevon D Fursa SR Malcolm AL . Intercoder Reliability and Validity of Webplotdigitizer in Extracting Graphed Data. Behav Modification (2017) 41(2):323–39. doi: 10.1177/0145445516673998 27760807

[32] Li L Zhao J Zhang F Fang F Shen Z Zhou Y . Changes of Intestinal Flora in Elderly Patients With Type 2 Diabetes Mellitus Complicated With Chronic Kidney Disease. Chin J Microecol (2021) 33(02):173–787. doi: 10.13381/j.cnki.cjm.202102009

[33] Tao S Li L Li L Liu Y Ren Q Shi M . Understanding the Gut-Kidney Axis Among Biopsy-Proven Diabetic Nephropathy, Type 2 Diabetes Mellitus and Healthy Controls: An Analysis of the Gut Microbiota Composition. Acta Diabetol (2019) 56(5):581–92. doi: 10.1007/s00592-019-01316-7 30888537

[34] Al-Obaide MA Singh R Datta P Salguero MV Vasylyeva TL . Gut Microbiota-Dependent Trimethylamine-N-Oxide and Inflammatory Biomarkers in Patients With Diabetic Nephropathy. J Am Soc Nephrol (2017) 28:208. doi: 10.3390/jcm6090086 PMC5615279 28925931

[35] Winther SA Henriksen P Vogt JK Hansen TH Ahonen L Suvitaival T . Gut Microbiota Profile and Selected Plasma Metabolites in Type 1 Diabetes Without and With Stratification by Albuminuria. Diabetologia (2020) 63(12):2713–24. doi: 10.1007/s00125-020-05260-y 32886190

[36] Li Y Yang M Sun B Zhou X Li G Li F . Correlation Analysis of Gut Microbia in Patients With Early and Advanced Type 2 Diabetic Kidney Disease. Chin J Clinical Rational Drug Use (2019) 12(7B):18–20. doi: 10.15887/j.cnki.13-1389/r.2019.20.009

[37] Li L Zhang F Fang F Shen Z Zhao J . Correlation Analysis of Distribution and Changes of Intestinal Microflora and Inflammatory Indexes in Patients With Dkd. Chin J Endocr Surg (2020) 14(6):507–10. doi: 10.3760/cma.j.cn.115807-20200708-00213

[38] Gradisteanu G Stoica R Petcu L Picu A Suceveanu A Salmen T . Microbiota Signatures in Type-2 Diabetic Patients With Chronic Kidney Disease - a Pilot Study. J Mind Med Sci (2019) 6(1):130–6. doi: 10.22543/7674.61.P130136

[39] Lin H Wen J . The Relationship Between Intestinal Microflora Disorder and Diabetic Nephropathy. J Pract Diabetol (2020) 16(05):125–6.

[40] Song D Mi Y Wang C . Patterns of Intestinal Flora Imbalance in Diabetic Kidney Disease and Type 2 Diabetes Based Upon High-Throughput Sequencing. J Clin Nephrol (2021) 21(11):887–94. doi: 10.3969/j.issn.1671-2390.2021.11.002

[41] Chun H Li L . Intestinal Microflora Diversity in Elderly T2dm Patients With Early Nephropathy. Chin J Microecol (2021) 33(8):916–9. doi: 10.13381/j.cnki.cjm.202108010

[42] Yaxian S . Analysis of Intestinal Flora of Type 2 Diabetic Suffered From Earlier Changes of Renal Function [Master]. Liaoning: Dalian Medical University (2016).

[43] Shade A . Diversity Is the Question, Not the Answer. ISME J (2017) 11(1):1–6. doi: 10.1038/ismej.2016.118 27636395 PMC5421358

[44] Valdes AM Walter J Segal E Spector TD . Role of the Gut Microbiota in Nutrition and Health. BMJ (Clinical Res ed) (2018) 361:k2179. doi: 10.1136/bmj.k2179 PMC6000740 29899036

[45] Subramaniam S Fletcher C . Trimethylamine N-Oxide: Breathe New Life. Br J Pharmacol (2018) 175(8):1344–53. doi: 10.1111/bph.13959 PMC5866995 28745401

[46] Seldin MM Meng Y Qi H Zhu W Wang Z Hazen SL . Trimethylamine N-Oxide Promotes Vascular Inflammation Through Signaling of Mitogen-Activated Protein Kinase and Nuclear Factor-Kb. J Am Heart Assoc (2016) 5(2):e002767. doi: 10.1161/jaha.115.002767 26903003 PMC4802459

[47] Sun G Yin Z Liu N Bian X Yu R Su X . Gut Microbial Metabolite Tmao Contributes to Renal Dysfunction in a Mouse Model of Diet-Induced Obesity. Biochem Biophys Res Commun (2017) 493(2):964–70. doi: 10.1016/j.bbrc.2017.09.108 28942145

[48] Shan Z Sun T Huang H Chen S Chen L Luo C . Association Between Microbiota-Dependent Metabolite Trimethylamine-N-Oxide and Type 2 Diabetes. Am J Clin Nutr (2017) 106(3):888–94. doi: 10.3945/ajcn.117.157107 28724646

[49] Tang WH Wang Z Li XS Fan Y Li DS Wu Y . Increased Trimethylamine N-Oxide Portends High Mortality Risk Independent of Glycemic Control in Patients With Type 2 Diabetes Mellitus. Clin Chem (2017) 63(1):297–306. doi: 10.1373/clinchem.2016.263640 27864387 PMC5659115

[50] Winther SA Øllgaard JC Tofte N Tarnow L Wang Z Ahluwalia TS . Utility of Plasma Concentration of Trimethylamine N-Oxide in Predicting Cardiovascular and Renal Complications in Individuals With Type 1 Diabetes. Diabetes Care (2019) 42(8):1512–20. doi: 10.2337/dc19-0048 PMC7082641 31123156

[51] Zhao X Zhang Y Guo R Yu W Zhang F Wu F . The Alteration in Composition and Function of Gut Microbiome in Patients With Type 2 Diabetes. J Diabetes Res (2020) 2020:8842651. doi: 10.1155/2020/8842651 33224990 PMC7673948

[52] Cinek O Kramna L Mazankova K Odeh R Alassaf A Ibekwe MU . The Bacteriome at the Onset of Type 1 Diabetes: A Study From Four Geographically Distant African and Asian Countries. Diabetes Res Clin Pract (2018) 144:51–62. doi: 10.1016/j.diabres.2018.08.010 30121305

[53] Chen S Henderson A Petriello MC Romano KA Gearing M Miao J . Trimethylamine N-Oxide Binds and Activates Perk to Promote Metabolic Dysfunction. Cell Metab (2019) 30(6):1141–51.e5. doi: 10.1016/j.cmet.2019.08.021 31543404

[54] Ma C Wickham ME Guttman JA Deng W Walker J Madsen KL . Citrobacter Rodentium Infection Causes Both Mitochondrial Dysfunction and Intestinal Epithelial Barrier Disruption in Vivo: Role of Mitochondrial Associated Protein (Map). Cell Microbiol (2006) 8(10):1669–86. doi: 10.1111/j.1462-5822.2006.00741.x 16759225

[55] Lee AS Gibson DL Zhang Y Sham HP Vallance BA Dutz JP . Gut Barrier Disruption by an Enteric Bacterial Pathogen Accelerates Insulitis in Nod Mice. Diabetologia (2010) 53(4):741–8. doi: 10.1007/s00125-009-1626-y 20012858

[56] Ramezani A Raj DS . The Gut Microbiome, Kidney Disease, and Targeted Interventions. J Am Soc Nephrol JASN (2014) 25(4):657–70. doi: 10.1681/asn.2013080905 PMC3968507 24231662

[57] Croxen MA Law RJ Scholz R Keeney KM Wlodarska M Finlay BB . Recent Advances in Understanding Enteric Pathogenic Escherichia Coli. Clin Microbiol Rev (2013) 26(4):822–80. doi: 10.1128/cmr.00022-13 PMC3811233 24092857

[58] Wada J Makino H . Innate Immunity in Diabetes and Diabetic Nephropathy. Nat Rev Nephrol (2016) 12(1):13–26. doi: 10.1038/nrneph.2015.175 26568190

[59] Wirth R Bódi N Maróti G Bagyánszki M Talapka P Fekete É . Regionally Distinct Alterations in the Composition of the Gut Microbiota in Rats With Streptozotocin-Induced Diabetes. PloS One (2014) 9(12):e110440. doi: 10.1371/journal.pone.0110440 25469509 PMC4254516

[60] Gülden E Chao C Tai N Pearson JA Peng J Majewska-Szczepanik M . Trif Deficiency Protects Non-Obese Diabetic Mice From Type 1 Diabetes by Modulating the Gut Microbiota and Dendritic Cells. J Autoimmun (2018) 93:57–65. doi: 10.1016/j.jaut.2018.06.003 29960834 PMC6108920

[61] Allin KH Tremaroli V Caesar R Jensen BAH Damgaard MTF Bahl MI . Aberrant Intestinal Microbiota in Individuals With Prediabetes. Diabetologia (2018) 61(4):810–20. doi: 10.1007/s00125-018-4550-1 PMC6448993 29379988

[62] Follador R Heinz E Wyres KL Ellington MJ Kowarik M Holt KE . The Diversity of Klebsiella Pneumoniae Surface Polysaccharides. Microbial Genomics (2016) 2(8):e000073. doi: 10.1099/mgen.0.000073 28348868 PMC5320592

[63] Hiippala K Kainulainen V Kalliomäki M Arkkila P Satokari R . Mucosal Prevalence and Interactions With the Epithelium Indicate Commensalism of Sutterella Spp. Front Microbiol (2016) 7:1706. doi: 10.3389/fmicb.2016.01706 27833600 PMC5080374

[64] Pendyala S Walker JM Holt PR . A High-Fat Diet Is Associated With Endotoxemia That Originates From the Gut. Gastroenterology (2012) 142(5):1100–.e2. doi: 10.1053/j.gastro.2012.01.034 PMC3978718 22326433

[65] Delzenne NM Cani PD Everard A Neyrinck AM Bindels LB . Gut Microorganisms as Promising Targets for the Management of Type 2 Diabetes. Diabetologia (2015) 58(10):2206–17. doi: 10.1007/s00125-015-3712-7 26224102

[66] Cole DK Bulek AM Dolton G Schauenberg AJ Szomolay B Rittase W . Hotspot Autoimmune T Cell Receptor Binding Underlies Pathogen and Insulin Peptide Cross-Reactivity. J Clin Invest (2016) 126(9):3626. doi: 10.1172/jci89919 PMC5004936 27525441

[67] Saad MJ Santos A Prada PO . Linking Gut Microbiota and Inflammation to Obesity and Insulin Resistance. Physiol (Bethesda Md) (2016) 31(4):283–93. doi: 10.1152/physiol.00041.2015 27252163

[68] Saurus P Kuusela S Lehtonen E Hyvönen ME Ristola M Fogarty CL . Podocyte Apoptosis Is Prevented by Blocking the Toll-Like Receptor Pathway. Cell Death Dis (2015) 6(5):e1752. doi: 10.1038/cddis.2015.125 25950482 PMC4669704

[69] Sawa Y Takata S Hatakeyama Y Ishikawa H Tsuruga E . Expression of Toll-Like Receptor 2 in Glomerular Endothelial Cells and Promotion of Diabetic Nephropathy by Porphyromonas Gingivalis Lipopolysaccharide. PloS One (2014) 9(5):e97165. doi: 10.1371/journal.pone.0097165 24835775 PMC4023930

[70] Greer RL Dong X Moraes AC Zielke RA Fernandes GR Peremyslova E . Akkermansia Muciniphila Mediates Negative Effects of Ifnγ on Glucose Metabolism. Nat Commun (2016) 7:13329. doi: 10.1038/ncomms13329 27841267 PMC5114536

[71] Depommier C Everard A Druart C Plovier H Van Hul M Vieira-Silva S . Supplementation With Akkermansia Muciniphila in Overweight and Obese Human Volunteers: A Proof-Of-Concept Exploratory Study. Nat Med (2019) 25(7):1096–103. doi: 10.1038/s41591-019-0495-2 PMC6699990 31263284

[72] Plovier H Everard A Druart C Depommier C Van Hul M Geurts L . A Purified Membrane Protein From Akkermansia Muciniphila or the Pasteurized Bacterium Improves Metabolism in Obese and Diabetic Mice. Nat Med (2017) 23(1):107–13. doi: 10.1038/nm.4236 27892954

[73] de la Cuesta-Zuluaga J Mueller NT Corrales-Agudelo V Velásquez-Mejía EP Carmona JA Abad JM . Metformin Is Associated With Higher Relative Abundance of Mucin-Degrading Akkermansia Muciniphila and Several Short-Chain Fatty Acid-Producing Microbiota in the Gut. Diabetes Care (2017) 40(1):54–62. doi: 10.2337/dc16-1324 27999002

[74] Shin NR Lee JC Lee HY Kim MS Whon TW Lee MS . An Increase in the Akkermansia Spp. Population Induced by Metformin Treatment Improves Glucose Homeostasis in Diet-Induced Obese Mice. Gut (2014) 63(5):727–35. doi: 10.1136/gutjnl-2012-303839 23804561

[75] Zhou H Zhao X Sun L Liu Y Lv Y Gang X . Gut Microbiota Profile in Patients With Type 1 Diabetes Based on 16s Rrna Gene Sequencing: A Systematic Review. Dis Markers (2020) 2020:3936247. doi: 10.1155/2020/3936247 32908614 PMC7474751

[76] Karlsson FH Tremaroli V Nookaew I Bergström G Behre CJ Fagerberg B . Gut Metagenome in European Women With Normal, Impaired and Diabetic Glucose Control. Nature (2013) 498(7452):99–103. doi: 10.1038/nature12198 23719380

[77] Christiansen CB Gabe MBN Svendsen B Dragsted LO Rosenkilde MM Holst JJ . The Impact of Short-Chain Fatty Acids on Glp-1 and Pyy Secretion From the Isolated Perfused Rat Colon. Am J Physiol Gastrointest Liver Physiol (2018) 315(1):G53–g65. doi: 10.1152/ajpgi.00346.2017 29494208

[78] Li L Lili R Hui Q Min W Xue W Xin S . Combination of Glp-1 and Sodium Butyrate Promote Differentiation of Pancreatic Progenitor Cells Into Insulin-Producing Cells. Tissue Cell (2008) 40(6):437–45. doi: 10.1016/j.tice.2008.04.006 18573514

[79] Yadav H Lee JH Lloyd J Walter P Rane SG . Beneficial Metabolic Effects of a Probiotic *Via* Butyrate-Induced Glp-1 Hormone Secretion. J Biol Chem (2013) 288(35):25088–97. doi: 10.1074/jbc.M113.452516 PMC3757173 23836895

[80] Khan D Vasu S Moffett RC Irwin N Flatt PR . Islet Distribution of Peptide Yy and Its Regulatory Role in Primary Mouse Islets and Immortalised Rodent and Human Beta-Cell Function and Survival. Mol Cell Endocrinol (2016) 436:102–13. doi: 10.1016/j.mce.2016.07.020 27465830

[81] Lafferty RA Flatt PR Irwin N . Established and Emerging Roles Peptide Yy (Pyy) and Exploitation in Obesity-Diabetes. Curr Opin Endocrinol Diabetes Obes (2021) 28(2):253–61. doi: 10.1097/med.0000000000000612 33395088

[82] Huang W Man Y Gao C Zhou L Gu J Xu H . Short-Chain Fatty Acids Ameliorate Diabetic Nephropathy *Via* Gpr43-Mediated Inhibition of Oxidative Stress and Nf- KB Signaling. Oxid Med Cell Longevity (2020) 2020:4074832. doi: 10.1155/2020/4074832 PMC7422068 32831998

[83] Kikuchi K Saigusa D Kanemitsu Y Matsumoto Y Thanai P Suzuki N . Gut Microbiome-Derived Phenyl Sulfate Contributes to Albuminuria in Diabetic Kidney Disease. Nat Commun (2019) 10(1):1835. doi: 10.1038/s41467-019-09735-4 31015435 PMC6478834

