# Supplementary material for: The Specific Alteration of Gut Microbiota in Diabetic Kidney Diseases—A Systematic Review and Meta-Analysis
Source: Front Immunol. 2022 Jun 17;13:908219. doi: 10.3389/fimmu.2022.908219 (PMC9248803; doi:10.3389/fimmu.2022.908219)
Supplement: Supplementary file 1 [file DataSheet_1.docx]

Supplementary Material

**Supplementary Table 1.** Systematic search detail.

**Supplementary Table 2.** Quality assessment of the included studies using the Newcastle-Ottawa Scale.

**Supplementary Table 3.** Alpha diversity and beta diversity between DKD and HC.

**Supplementary Table 4.** Differentially abundant microbial taxa.

**Supplementary Table 5.** Intestinal microbiota metabolites and other markers (Intestinal permeability markers, inflammatory markers, endothelial dysfunction markers).

**Supplementary Table 6.** Functional characterizations of gut microbiota in patients with DKD.

**Supplementary Figure 1.** Publication bias assessment for the alpha diversity mete-analyses.

**Funnel plots assessing publication bias. A. Chao1, B. ACE, C. Shannon, D. Simpson, E. PD_whole_tree.**

**Supplementary Table 1. Systematic search detail.**

**1.1 PubMed**

| **Sequence** | **Search** | **Hits** |
| --- | --- | --- |
| 1 | ("Diabetic Nephropathies"[Mesh]) OR (((((((((((((((((Nephropathies, Diabetic[Title/Abstract]) OR (Nephropathy, Diabetic[Title/Abstract])) OR (Diabetic Nephropathy[Title/Abstract])) OR (Diabetic Kidney Disease[Title/Abstract])) OR (Diabetic Kidney Diseases[Title/Abstract])) OR (Kidney Disease, Diabetic[Title/Abstract])) OR (Kidney Diseases, Diabetic[Title/Abstract])) OR (Diabetic Glomerulosclerosis[Title/Abstract])) OR (Glomerulosclerosis, Diabetic[Title/Abstract])) OR (Intracapillary Glomerulosclerosis[Title/Abstract])) OR (Nodular Glomerulosclerosis[Title/Abstract])) OR (Glomerulosclerosis, Nodular[Title/Abstract])) OR (Kimmelstiel-Wilson Syndrome[Title/Abstract])) OR (Kimmelstiel Wilson Syndrome[Title/Abstract])) OR (Syndrome, Kimmelstiel-Wilson[Title/Abstract])) OR (Kimmelstiel-Wilson Disease[Title/Abstract])) OR (Kimmelstiel Wilson Disease[Title/Abstract])) | 36,649 |
| 2 | ("Microbiota"[Mesh]) OR ((((((((((((((((Microbiotas[Title/Abstract]) OR (Microbial Community[Title/Abstract])) OR (Community, Microbial[Title/Abstract])) OR (Microbial Communities[Title/Abstract])) OR (Microbial Community Composition[Title/Abstract])) OR (Community Composition, Microbial[Title/Abstract])) OR (Composition, Microbial Community[Title/Abstract])) OR (Microbial Community Compositions[Title/Abstract])) OR (Microbial Community Structure[Title/Abstract])) OR (Community Structure, Microbial[Title/Abstract])) OR (Microbial Community Structures[Title/Abstract])) OR (Microbiome[Title/Abstract])) OR (Microbiomes[Title/Abstract])) OR (Human Microbiome[Title/Abstract])) OR (Human Microbiomes[Title/Abstract])) OR (Microbiome, Human[Title/Abstract])) | 101,865 |
| 3 | 1 AND 2 | 56 |

**1.2 Web of science**

| **Sequence** | **Search** | **Hits** |
| --- | --- | --- |
| 1 | (diabetic Nephropathy OR Nephropathies, Diabetic OR Nephropathy, Diabetic OR Diabetic Nephropathy OR Diabetic Kidney Disease OR Diabetic Kidney Diseases OR Kidney Disease, Diabetic OR Kidney Diseases, Diabetic OR Diabetic Glomerulosclerosis OR Glomerulosclerosis, Diabetic OR Intracapillary Glomerulosclerosis OR Nodular Glomerulosclerosis OR Glomerulosclerosis, Nodular OR Kimmelstiel-Wilson Syndrome OR Kimmelstiel Wilson Syndrome OR Syndrome, Kimmelstiel-Wilson OR Kimmelstiel-Wilson Disease OR Kimmelstiel Wilson Disease) | 75,999 |
| 2 | (Microbiota OR Microbiotas OR Microbial Community OR Community, Microbial OR Microbial Communities OR Microbial Community Composition OR Community Composition, Microbial OR Composition, Microbial Community OR Microbial Community Compositions OR Microbial Community Structure OR Community Structure, Microbial OR Microbial Community Structures OR Microbiome OR Microbiomes OR Human Microbiome OR Human Microbiomes OR Microbiome, Human) | 261,484 |
| 3 | 1 AND 2 | 223 |

**1.3 Cochrane**

| **Sequence** | **Search** | **Hits** |
| --- | --- | --- |
| 1 | MeSH descriptor: [Diabetic Nephropathies] explode all trees | 1515 |
| 2 | (diabetic Nephropathy):ab,ti,kw OR (Nephropathies, Diabetic):ab,ti,kw OR (Nephropathy, Diabetic):ab,ti,kw OR (Diabetic Nephropathy):ab,ti,kw OR (Diabetic Kidney Disease):ab,ti,kw OR (Diabetic Kidney Diseases):ab,ti,kw OR (Kidney Disease, Diabetic):ab,ti,kw OR (Kidney Diseases, Diabetic):ab,ti,kw OR (Diabetic Glomerulosclerosis):ab,ti,kw OR (Glomerulosclerosis, Diabetic):ab,ti,kw OR (Intracapillary Glomerulosclerosis):ab,ti,kw OR (Nodular Glomerulosclerosis):ab,ti,kw OR (Glomerulosclerosis, Nodular):ab,ti,kw OR (Kimmelstiel-Wilson Syndrome):ab,ti,kw OR (Kimmelstiel Wilson Syndrome):ab,ti,kw OR (Syndrome, Kimmelstiel-Wilson):ab,ti,kw OR (Kimmelstiel-Wilson Disease):ab,ti,kw OR (Kimmelstiel Wilson Disease):ab,ti,kw | 6117 |
| 3 | 1 OR 2 | 6117 |
| 4 | MeSH descriptor: [Microbiota] explode all trees | 1073 |
| 5 | (microbiota):ab,ti,kw OR (Microbiotas):ab,ti,kw OR (Microbial Community):ab,ti,kw OR (Community, Microbial):ab,ti,kw OR (Microbial Communities):ab,ti,kw OR (Microbial Community Composition):ab,ti,kw OR (Community Composition, Microbial):ab,ti,kw OR (Composition, Microbial Community):ab,ti,kw OR (Microbial Community Compositions):ab,ti,kw OR (Microbial Community Structure):ab,ti,kw OR (Community Structure, Microbial):ab,ti,kw OR (Microbial Community Structures):ab,ti,kw OR (Microbiome):ab,ti,kw OR (Microbiomes):ab,ti,kw OR (Human Microbiome):ab,ti,kw OR (Human Microbiomes):ab,ti,kw OR (Microbiome, Human):ab,ti,kw | 7747 |
| 6 | 4 or 5 | 7749 |
| 7 | 3 AND 6 | 13 |

**1.4 Embase**

| **Sequence** | **Search** | **Hits** |
| --- | --- | --- |
| 1 | 'Diabetic Nephropathy'/exp | 48,960 |
| 2 | 'Diabetic Nephropathy':ab,ti OR 'Nephropathies, Diabetic':ab,ti OR 'Nephropathy, Diabetic':ab,ti OR 'Diabetic Nephropathy':ab,ti OR 'Diabetic Kidney Disease':ab,ti OR 'Diabetic Kidney Diseases':ab,ti OR 'Kidney Disease, Diabetic':ab,ti OR 'Kidney Diseases, Diabetic':ab,ti OR 'Diabetic Glomerulosclerosis':ab,ti OR 'Glomerulosclerosis, Diabetic':ab,ti OR 'Intracapillary Glomerulosclerosis':ab,ti OR 'Nodular Glomerulosclerosis':ab,ti OR 'Glomerulosclerosis, Nodular':ab,ti OR 'Kimmelstiel-Wilson Syndrome':ab,ti OR 'Kimmelstiel Wilson Syndrome':ab,ti OR 'Syndrome, Kimmelstiel-Wilson':ab,ti OR 'Kimmelstiel-Wilson Disease':ab,ti OR 'Kimmelstiel Wilson Disease':ab,ti | 33,529 |
| 3 | 1 OR 2 | 54,530 |
| 4 | 'Microbiota'/exp | 155,739 |
| 5 | 'Microbiotas':ab,ti OR 'Microbial Community':ab,ti OR 'Community, Microbial':ab,ti OR 'Microbial Communities':ab,ti OR 'Microbial Community Composition':ab,ti OR 'Community Composition, Microbial':ab,ti OR 'Composition, Microbial Community':ab,ti OR 'Microbial Community Compositions':ab,ti OR 'Microbial Community Structure':ab,ti OR 'Community Structure, Microbial':ab,ti OR 'Microbial Community Structures':ab,ti OR 'Microbiome':ab,ti OR 'Microbiomes':ab,ti OR 'Human Microbiome':ab,ti OR 'Human Microbiomes':ab,ti OR 'Microbiome, Human':ab,ti | 79,707 |
| 6 | 4 OR 5 | 185,891 |
| 7 | 3 AND 6 | 184 |

**1.5 CBM**

| **Sequence** | **Search** | **Hits** |
| --- | --- | --- |
| 1 | (" glomerulosclerosis") OR (" diabetic glomerulosclerosis ") OR (" diabetic nephropathy ") | 78,072 |
| 2 | (" gastrointestinal microbiome ") OR (" the human microbiome") OR (" microbiome;") OR (" microbial community ") OR (" microbiota") | 100,260 |
| 3 | 1 AND 2 | 69 |

CBM, Chinese Biomedical Databases.

**1.6 CNKI**

| **Sequence** | **Search** | **Hits** |
| --- | --- | --- |
| 1 | glomerulosclerosis OR diabetic glomerulosclerosis OR diabetic nephropathy | 57,092 |
| 2 | gastrointestinal microbiome OR the human microbiome OR microbiome OR microbial community OR microbiota | - |
| 3 | 2 is retrieved in the result of 1 | 88 |

CNKI, China National Knowledge Internet.

**Supplementary Table 2.** **Quality assessment of the included studies using the Newcastle-Ottawa Scale.**

| **Study** | **Selection** | | | **Comparability** | | | **Ascertainment exposure** | | | **Score** |
| --- | --- | --- | --- | --- | --- | --- | --- | --- | --- | --- |
|  | **1** | **2** | **3** | **4** | **5A** | **5B** | **6** | **7** | **8** |  |
| Sibei Tao et al.2019 | * | * | * | * | * | * | * |  | * | 8* |
| Bao Xuguang et al.2019 | * | * | * | * | * |  | * |  | * | 7* |
| Feng Chunnian et al.2020 | * | * | * | * | * |  | * |  | * | 7* |
| Li Lei et al.2021 | * | * | * | * | * | * | * |  | * | 8* |
| Li Lei et al.2020 | * | * | * | * | * | * | * |  | * | 8* |
| Lin Hao et al.2020 |  | * | * | * | * |  | * |  | * | 6* |
| Song Dandan et al.2021 | * | * | * | * | * |  | * |  | * | 7* |
| Chun Huan et al.2021 | * | * | * | * | * |  | * |  | * | 7* |
| Sun Ya xian et al.2016 | * | * | * | * |  |  | * |  | * | 6* |
| Li Ya et al.2019 | * | * | * |  | * |  | * |  | * | 6* |
| Xin Xiaohong et al.2021 | * | * | * | * | * |  | * |  | * | 7* |
| Xi Du et al.2021 | * | * | * | * | * | * | * |  | * | 8* |
| Mohammed A. I. Al-Obaide et al.2017 | * | * | * | * | * |  | * | * | * | 8* |
| MARIA V. SALGUERO et al.2019 | * | * | * | * | * |  | * |  | * | 7* |
| Gratiela P. Gradisteanu et al.2019 | * | * | * | * |  |  | * |  | * | 6* |
| Signe A. Winther et al.2020 | * | * | * |  | * |  | * | * | * | 7* |

**Supplementary Table 3. Alpha diversity and beta diversity between DKD and HC.**

| **Study** | **Alpha-diversity Index** | **Conclusion** | **Beta-diversity Index** | **Conclusion** |
| --- | --- | --- | --- | --- |
| Sibei Tao et al.2019 | Sobs, Shannon, Simpson, ACE, Chao | The differences of diversity were not significant, different richness were found. | PCoA | Distinct |
| Bao Xuguang et al.2019 | PD_whole_tree | Both richness and diversity decreased, whereas diversity was not statistically different. | PCoA | Distinct |
| Feng Chunnian et al.2020 | PD_whole_tree | Decrease | PCoA | Distinct |
| Li Lei et al.2021 | N/A | N/A | N/A | N/A |
| Li Lei et al.2020 | N/A | N/A | N/A | N/A |
| Lin Hao et al.2020 | N/A | N/A | N/A | N/A |
| Song Dandan et al.2021 | Observed species, Chao1, Shannon, Simpson | Richness decreased and diversity was not statistically different. | PCoA | Distinct |
| Chun Huan et al.2021 | ACE, Chao1, Shannon, Simpson | Similar | N/A | N/A |
| Sun Ya xian et al.2016 | PCR-DGGE | Decrease | PCA | Distinct |
| Li Ya et al.2019 | N/A | N/A | N/A | N/A |
| Xin Xiaohong et al.2021 | Shannon | Increase | PCoA | Distinct |
| Xi Du et al.2021 | Observed, Chao1, ACE, Fisher index，Shannon, Simpson | Decrease | PCA, PCoA, NMDS | Distinct |
| Mohammed A. I. Al-Obaide et al.2017 | N/A | N/A | N/A | N/A |
| MARIA V. SALGUERO et al.2019 | N/A | N/A | N/A | N/A |
| Gratiela P. Gradisteanu et al.2019 | N/A | N/A | N/A | N/A |
| Signe A. Winther et al.2020 | Observed genera, Shannon, Simpson | Similar | PCoA | Distinct |

DKD, diabetic kidney disease; HC, healthy controls; PD_whole_tree, phylogenetic diversity whole tree; PCR-DEEG, polymerase chain reaction-denaturing gradient gel electrophoresis; PCoA, principal coordinate analysis; PCA, principal component analysis; NMDS, nonmetric multidimensional scaling; N/A, not available.

**Supplementary Table 4. Differentially Abundant Microbial Taxa.**

| **Study** | **Phylum level** | | **Class level** | | **Order level** | | **Family level** | | **Genus level** | |
| --- | --- | --- | --- | --- | --- | --- | --- | --- | --- | --- |
|  | **Higher** | **Lower** | **Higher** | **Lower** | **Higher** | **Lower** | **Higher** | **Lower** | **Higher** | **Lower** |
| Sibei Tao et al.2019 | Proteobacteria | Firmicutes | N/A | N/A | N/A | N/A | Coriobacteriaceae | Prevotellaceae | Prevotella_9 | Escherichia-Shigella |
| Bao Xuguang et al.2019 | Synergistetes | Firmicutes, Cyanobacteria | N/A | N/A | N/A | N/A | Synergistaceae,  Coriobacteriaceae,  Enterobacteriaceae,  Lactobacteriaceae,  Veillonellaceae | Lachnospiraceae | Lactobacillus, Haemophilus, Enterococcus, Slackia, Anaerotruncus | Lachnospira, Turicibacter, Faecalibacterium, Roseburia, Coprococcus |
| Feng Chunnian et al.2020 | Bacteroidetes, Actinobacteria | Firmicutes | N/A | N/A | N/A | N/A | N/A | N/A | Lachnospira, Rothia, Coprococcus | Bifidobacterium |
| Li Lei et al.2021 | N/A | N/A | N/A | N/A | N/A | N/A | N/A | N/A | E. coli | Bifidobacteria, Bacillus |
| Li Lei et al.2020 | N/A | N/A | N/A | N/A | N/A | N/A | N/A | N/A | E. coli | Bifidobacteria, ture bacteria |
| Lin Hao et al.2020 | N/A | N/A | N/A | N/A | N/A | N/A | N/A | N/A | Coprococcus | Proteus, Lactobacillus |
| Song Dandan et al.2021 | N/A | Firmicutes | N/A | N/A | Orynebacteriales | N/A | N/A | N/A | Eisenbergiella, Ralstonia, Intestinimonas, Eubacterium_fissicatena_group | N/A |
| Chun Huan et al.2021 | Bacteroidetes | Firmicutes | N/A | N/A | N/A | N/A | N/A | N/A | N/A | N/A |
| Sun Ya xian et al.2016 | N/A | N/A | N/A | N/A | N/A | N/A | N/A | N/A | Flavonifractor plauti | prevotella, Lactobacillus |
| Li Ya et al.2019 | N/A | N/A | N/A | N/A | N/A | N/A | N/A | N/A | Coprococcus | Brautia |
| Xin Xiaohong et al.2021 | N/A | N/A | N/A | N/A | N/A | N/A | N/A | N/A | Citrobacter, Escherichia，Hungatella, Ervsipelatoclostridium, Klebsiella, Akkermansia, Lachnoclostridium | Prevotella, Rothia |
| Xi Du et al.2021 | Actinobacteria | N/A | Actinobacteria, Bacilli, Coriobacteriia, Negativicutes | Alphaproteobacteria,  Clostridia | Betaproteobacteriales,  Bifidobacteriales, Coriobacteriales, Lactobacillales, Selenomonadales | Chitinophagales, Clostridiales, Rhizobiales, Xanthomonadales | Atopobiaceae, Bifidobacteriaceae, Burkholderiaceae, Lactobacillaceae, Streptococcaceae, Tannerellaceae, Veillonellaceae | Chitinophagaceae, Lachnospiraceae, Rhodanobacteraceae | Acidaminococcus, Lactobacillus, Megasphaera, Mitsuokella, Olsenella, Prevotella_7, Sutterella | Lachnoclostridium, Roseburia, Tyzzerella_3 |
| Mohammed A. I. Al-Obaide et al.2017 | N/A | N/A | N/A | N/A | N/A | N/A | N/A | N/A | Anaerococcus, Clostridium, Desulfitobacter, Enterococcus, Streptococcus, Desulfovibrio, Enterobacter, Escherichia, Klebsiella,  Proteus, Pseudomonas, Acinetobacter, Citrobacter, Lactobacillus | Bifidobacteriums |
| MARIA V. SALGUERO et al.2019 | Proteobacteria, Verrucomicrobia, Fusobacteria | N/A | N/A | N/A | N/A | N/A | Moraxellaceae,  Xanthomonadaceae,  Enterobacteriaceae,  Pasteurellaceae,  Alcaligenaceae,  Verrucomicrobiaceae, Fusobacteriacea,  Leptotrichiacene | N/A | Acinetobacter, Enhydrobacter, Stenotrophomonas, Citrobacter, Cronobacter, Enterobacter, Erwinia, Escherichia, Klebsiella, Pantoea, Proteus, Serratia, Trabulsiella, Actinobacillus, Achromobacter, Aggregatibacter, Sutterella, Akkermansia, Fusobacterium, Leptotrichia | N/A |
| Gratiela P. Gradisteanu et al.2019 | N/A | N/A | N/A | N/A | N/A | N/A | Enterobacteriaceae | N/A | Turicibacter sp | N/A |
| Signe A. Winther et al.2020 | N/A | N/A | N/A | Bacterodia | N/A | Selenomonadales | N/A | N/A | Anaerostipes | CHKCI002, Allisonella, [Bacteroides] pectinophilus group |

**N/A, not available.**

**Supplementary Table 5. Intestinal microbiota metabolites and other markers（Intestinal permeability markers, inflammatory markers, endothelial dysfunction markers）.**

| **Study** | **Type of samples** | **Index** | **Conclusion** |
| --- | --- | --- | --- |
| Bao Xuguang et al.2019 | Serum | IL-6, CRP | The serum levels of IL-6 and CRP in T2D and DKD were significantly higher than those in HC. |
| Feng Chunnian et al.2020 | Serum | Hs-CRP, IL-6 | The serum levels of Hs-CRP and IL-6 in T2D and DKD were significantly higher than those in HC, the levels of the above indexes in DKD were higher than those in T2D. |
| Li Lei et al.2020 | Serum | TNF-α, IL-6, IL-8, Hs-CRP | The serum levels of TNF-α, IL-6, Hs-CRP: DKD > T2D > HC, and the difference was statistically significant. The level of IL-8 in DKD and T2D group was significantly higher than that in controls. |
| Mohammed A. I. Al-Obaide et al.2017 | Serum | TMAO, ET-1, CRP, TNF-α, Zo, LPS | The serum levels of TMAO in patients with DKD were significantly higher than controls. TMAO showed a positive correlation with Zo, LPS, inflammatory, and endothelial dysfunction biomarkers. The zonulin (Zo) measurements, the two inflammatory markers TNFα and IL-6, and the endothelial dysfunction marker ET-1 showed an increased level in DKD, compared to healthy subjects. LPS was higher in DKD compared with healthy subjects. LPS, CRP levels showed no significant difference between DKD and healthy subjects. |
| MARIA V. SALGUERO et al.2019 | Serum | ET-1, CRP, TNF-α, IL6, LPS | The serum levels of CRP、IL6、TNFα and LPS were significantly increased in patients with DKD compared with controls. |
| Signe A. Winther et al.2020 | Plasma | Tryptophan, L-citrulline, indoxyl sulphate | Compared with HC, patients with type 1 diabetes with macroalbuminuria had lower concentrations of tryptophan and higher concentrations of L-citrulline. The higher plasma concentrations of indoxyl sulphate in individuals with type 1 diabetes with macroalbuminuria compared with micro- and normoalbuminuria and an inverse correlation with eGFR. There was no significant difference between type 1 diabetes and healthy controls. |

IL-6, interleukin-6; CRP, C-reactive protein; Hs-CRP, high-sensitivity C-reactive protein; TNF-α, tumor necrosis factor-α; IL-8, interleukin-8; TMAO, Trimethylamine-N-oxide; ET-1: endothelin-1; Zo: zonulin, gut permeability marker; LPS, lipopolysaccharide; DKD, Diabetic kidney disease; HC, Healthy Controls; T2D, Type 2 diabetes.

**Supplementary Table 6. Functional characterizations of gut microbiota in patients with DKD.**

| **Study** | **Methods** | **Conclusion** |
| --- | --- | --- |
| Bao Xuguang et al.2019 | PICRUSt, KEGG | The functional changes of T2D and DKD were more consistent. The functions of intestinal gluconeogenesis, glycosphingolipid biosynthesis, glycosyltransferase, tricarboxylic acid cycle, tryptophan metabolism, valine degradation, leucine and isoleucine, lipopolysaccharide biosynthesis protein and biosynthesis of lipopolysaccharides, glycan biosynthesis, degradation of aminobenzoic acid esters, bacterial toxin, glycosaminoglycan degradation, transport and metabolism of inorganic substances, cell movement and secretion, Staphylococcus aureus infection, propionic acid metabolism, Vibrio cholera infection, metabolism of ascorbic acid and uronic acid, phosphate transport system and geraniol metabolism were significantly enhanced in DKD compared with T2D. The functions of thiamine metabolism, porphyrin metabolism, lysine biosynthesis, phenylalanine biosynthesis, RNA transport, calcium signal pathway, phenylalanine biosynthesis, tyrosine biosynthesis, tryptophan biosynthesis, and methane metabolism were decreased significantly in DKD. |
| Xin Xiaohong et al.2021 | KEGG | The metabolic pathway of tyrosine is high expression and the metabolic pathway of short chain fatty acids is the low expression in DN |

KEGG, Kyoto Encyclopedia of Genes and Genomes; PICRUSt, Phylogenetic Investigation of Communities by Reconstruction of Unobserved States; DKD, Diabetic kidney disease; T2D, Type 2 diabetes; DN, Diabetic nephropathy confirmed by renal biopsy.

**Supplementary Figure 1. Publication bias assessment for the alpha diversity mete-analyses.**


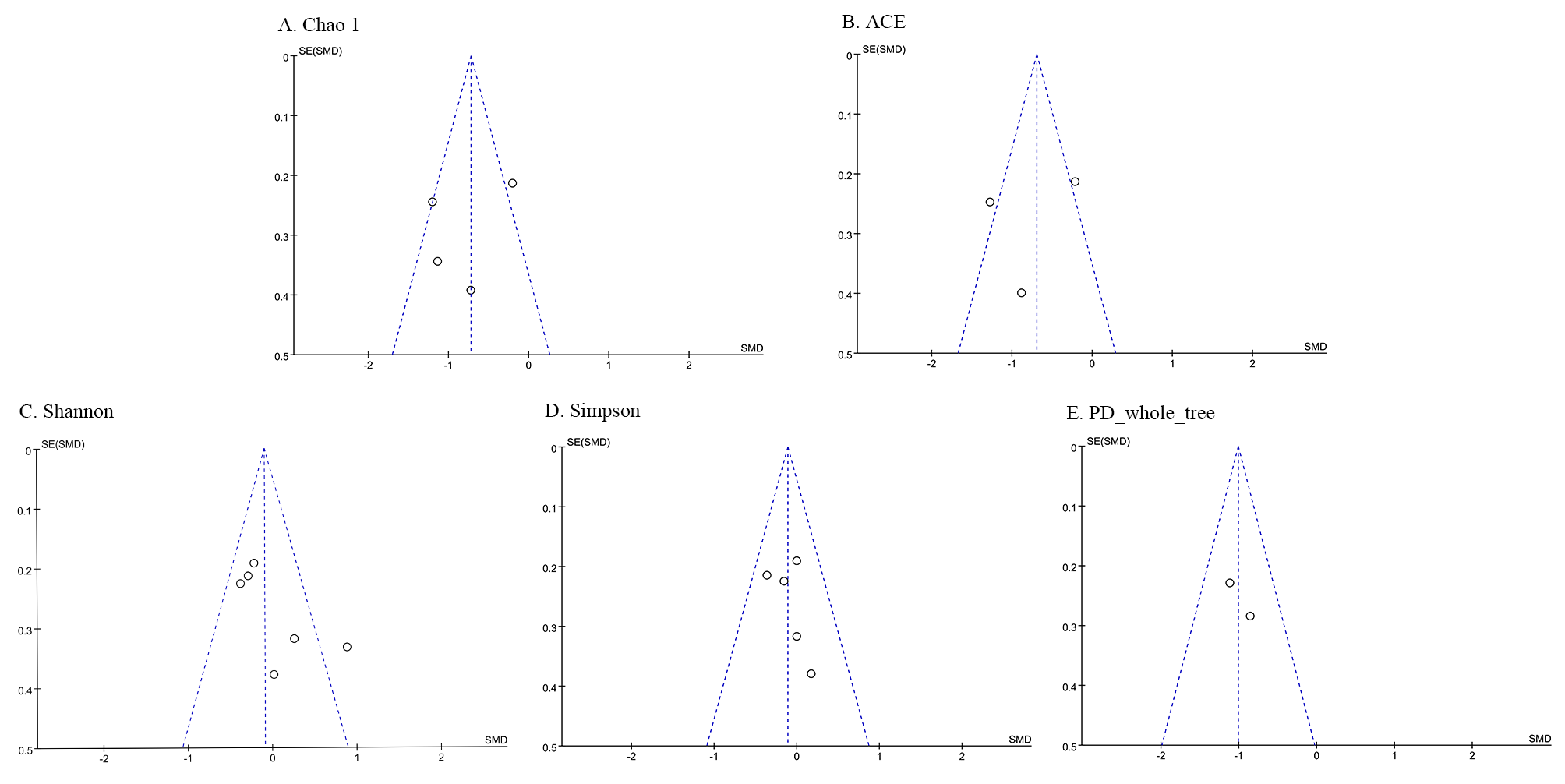


**Funnel plots assessing publication bias. A. Chao1, B. ACE, C. Shannon, D. Simpson, E. PD_whole_tree.**
